# Sepsis – it is all about the platelets

**DOI:** 10.3389/fimmu.2023.1210219

**Published:** 2023-06-07

**Authors:** Dermot Cox

**Affiliations:** School of Pharmacy and Biomolecular Sciences, Royal College of Surgeons in Ireland, Dublin, Ireland

**Keywords:** platelets, sepsis, innate immunity, thrombosis, anti-platelet agents

## Abstract

Sepsis is accompanied by thrombocytopenia and the severity of the thrombocytopenia is associated with mortality. This thrombocytopenia is characteristic of disseminated intravascular coagulation (DIC), the sepsis-associated coagulopathy. Many of the pathogens, both bacterial and viral, that cause sepsis also directly activate platelets, which suggests that pathogen-induced platelet activation leads to systemic thrombosis and drives the multi-organ failure of DIC. In this paper we review the mechanisms of platelet activation by pathogens and the evidence for a role for anti-platelet agents in the management of sepsis.

## Introduction

The development of a circulatory system was critical in the evolution of complex organisms. However, this also created the vulnerability that loss of blood due to injury could be fatal to the organism. Thus, it was essential that a system that could limit blood-loss was also developed. Furthermore, as blood is also a very fertile environment for the growth of bacteria, especially after a trauma, there was a need to develop a system to fight infection. In the case of primitive organisms such as the Horseshoe crab, a single cell – the haematocyte – fulfilled both these requirements. This cell could respond to bacteria and fight the infection. Once stimulated they also clumped together sealing any leak. Thus, the haematocyte mediated both the immune and haemostatic responses (1).

As organisms became more complex, so too did the regulatory systems. This simple haematocyte evolved into multiple specialised cells such as leucocytes and monocytes. One specific cell type – the megakaryocyte - became the sole cell type responsible for haemostasis. While not directly involved in haemostasis the megakaryocyte fragments into platelets, which are the key regulator and mediator of haemostasis.

Since megakaryocytes evolved from the haematocyte it is not surprising that not only did they acquire the haemostatic properties of haematocytes, they also retained some of the immune functions of the haematocyte. Thus, as well as being mediators of haemostasis platelets are also part of the innate immune system.

The immune and haemostatic response to injury were conventionally considered as distinct systems. However, recently it has become clear that the two are intimately linked. The role of platelets in mediating the innate immune response to infection is known as immunothrombosis (2) and is a normal physiological response to infection. Thrombus formation is a complex process that also involves cells of the innate immune system and the role of the innate immune response in thrombus formation is known as thrombo-inflammation (3). Thus, infection activates the innate immune system and if this innate immune response persists (either because the pathogen is resistant or if it is an autoimmune response) it results in enhanced thrombus formation (4, 5). Platelets are the first responders to trauma (in part due to their high concentration in plasma) and their activation leads to recruitment of immune cells to the site of injury as well as the formation of a clot. Thus, platelet activation acts to both prevent blood-loss and to sterilize the site of injury.

## Platelets and thrombosis

Platelets can be considered to have multiple distinct functions that are often, but not necessarily, connected. These are adhesion, aggregation, secretion, and platelet-leucocyte complex formation.

Platelets are highly responsive to many of the components found at the site of injury. Thus, they have receptors for collagen, fibrinogen and von Willebrand factor (vWF) all of which are to be found on the damaged blood vessel. The primary function of the interaction with these ligands is to immobilise the platelet to the damaged vessel under both low shear (venous) conditions (collagen and fibrinogen receptors) and high shear (arterial) conditions (vWF receptors) (6). Platelets can also bind to bacteria that have attached to a surface. Platelet adhesion mediated by these receptors usually results in platelet activation facilitating thrombus formation, while also ensuring that thrombus formation is restricted to the site of injury.

Platelets also express receptors for soluble ligands. These are all G-protein coupled receptors and respond to ligands such as ADP, adrenaline, and thrombin. Platelet activation by these ligands is important in recruiting platelets to the site of injury and growing the thrombus (7).

Once activated, platelets aggregate forming a thrombus, but they also secrete the content of their granules. Platelets contain multiple granule types including alpha- granules, dense granules, and lysosomes. This platelet secretome is rich in bioactive molecules including over 2,000 proteins (8), small molecules such as ADP and serotonin and polyphosphates (9). While the platelet secretome plays a role in thrombosis it is primarily involved in the non-thrombotic roles of platelets.

While platelet-platelet interactions are a critical property of platelets, activated platelets can also bind to leucocytes and endothelial cells. This interaction can modify the function of the target cell.

## Platelets and the innate immune system

While platelets play a critical role in haemostasis it has become clear that they also play a role in the innate immune system. As there are many dedicated immune cell types any immune function of platelets is likely redundant. However, as platelets are the first responders to a cut (a primary cause of infection), they are perfectly placed to help sterilise the wound and to coordinate the immune response to the injury.

There is strong evidence to suggest that this happens clinically. Serious infections are associated with thrombocytopenia (10) and this thrombocytopenia is associated with outcome. Thus, in sepsis the extent of thrombocytopenia is associated with severity of disease and outcome (11–13). Furthermore, in severe viral infections (14) such as Dengue (15), COVID-19 (16), Hantavirus (17), Hepatitis B (18) and mononucleosis (19) the severity of the thrombocytopenia is associated with outcome.

The cause of this thrombocytopenia is unclear, and it has been proposed that it could be due to suppression of platelet production by the megakaryocytes as a result of infection. There is certainly evidence that megakaryocytes can be infected by viruses such as Dengue virus (DENV) and influenza virus (20). However, the result of this infection is complex. Megakaryocyte infection by DENV has been shown to reduce megakaryocyte levels which would lead to decreased platelet production (21). However, inflammation has also been shown to increase platelet function and an increase in platelet count has been seen in the initial response to COVID-19 (22) and increased levels of IL-1 and CCL5 increase platelet production by around 50% (23, 24). Ultimately, changes in platelet synthesis are unlikely to be relevant as the lifespan of a platelet is approximately 10 days and even if infection entirely shut down platelet production it would take 9 days for severe thrombocytopenia to occur, while in sepsis it occurs rapidly. Furthermore, while thrombocytopenia would create a risk of bleeding it is unlikely to be involved in the pathogenesis of the infection and would only be a biomarker of outcome.

Thus, it is likely that infection-associated thrombocytopenia is due to platelet activation as this is likely to be rapid in onset and the resultant large-scale thrombosis would have pathological consequences. This level of platelet activation will result in extensive thrombosis throughout the circulation. This is in fact what happens in a condition known as disseminated intravascular coagulation (DIC). DIC is a coagulopathy that is associated with severe infections such as sepsis and COVID-19 and is characterised by thrombocytopenia, platelet activation and systemic thrombosis (25). If this thrombosis occurs in the microvasculature of multiple organs, it will lead to ischemic damage. As this ischemic damage expands, this leads to the multi-organ failure of severe sepsis.

The critical question is how the platelet activation arises. One possibility is that platelets are innocent bystanders. Infection leads to a highly pro-inflammatory environment that in turn could lead to platelet activation. Evidence for this is that platelets can be activated by some cytokines including IL-6 and IL-8 (26). Dengue virus has been shown to induce IL-1β production which in turn induces iNOS in platelets (27). Another possibility is that platelets may interact with inflammed/infected endothelial cells leading to their activation (28). It is important to note that not all platelet activation is the same. Platelets have been shown to undergo a form of activation known as pyroptosis (29), which can be considered to be a cross between apoptosis (membrane blebbing and caspase activation) and necrosis (cell swelling and lysis) (30). This is a pro-inflammatory response that involves inflammasome formation. Pyroptosis is induced in platlelets by circulating S100A8/A9 (29).

Infection can lead to thrombin generation either through activation of the contact system or generation of tissue factor. As well as causing the formation of fibrin clots this thrombin will also activate platelets. However, this is unlikely to be clinically relevant and activated protein C (aPC) is a thrombin inhibitor and was introduced for the treatment of sepsis (31), however, it was ultimately removed from the market due to the lack of benefit (32). Furthermore, while the use of anti-thrombins have some benefit in both sepsis (33) and COVID-19 (34) their use has been disappointing as DIC continues to be a major problem. This suggests that while thrombin generation may occur during inflammation/infection it is not the major driver of DIC in humans not withstanding the benefit of these agents in animal models of DIC.

Both thrombosis and inflammation are closely intertwined and this connection is bi-directional, with inflammation leading to increased thrombosis and vice-versa, and is known as immunothrombosis (35). While the production of pro-inflammatory cytokines or thrombin generation may play a minor role in DIC it is clear that platelet activation in response to infection is unikely to be incidental and is more likely to be a direct response to infection.

## Pathogen interaction with platelet haemostasis receptors

Considering that platelets are the first responders to injury it is reasonable that they would respond directly to any infecting pathogen. It is no conicidence that nearly all of the bacteria that are associated with sepsis have been shown to induce platelet aggregation when added to platelet-rich plasma (PRP) which presumably represents a direct interaction between pathogen and platelets. Furthermore, bacteria such as *S. aureus* have multiple different interactions with platelets (36).

**Figure 1:**
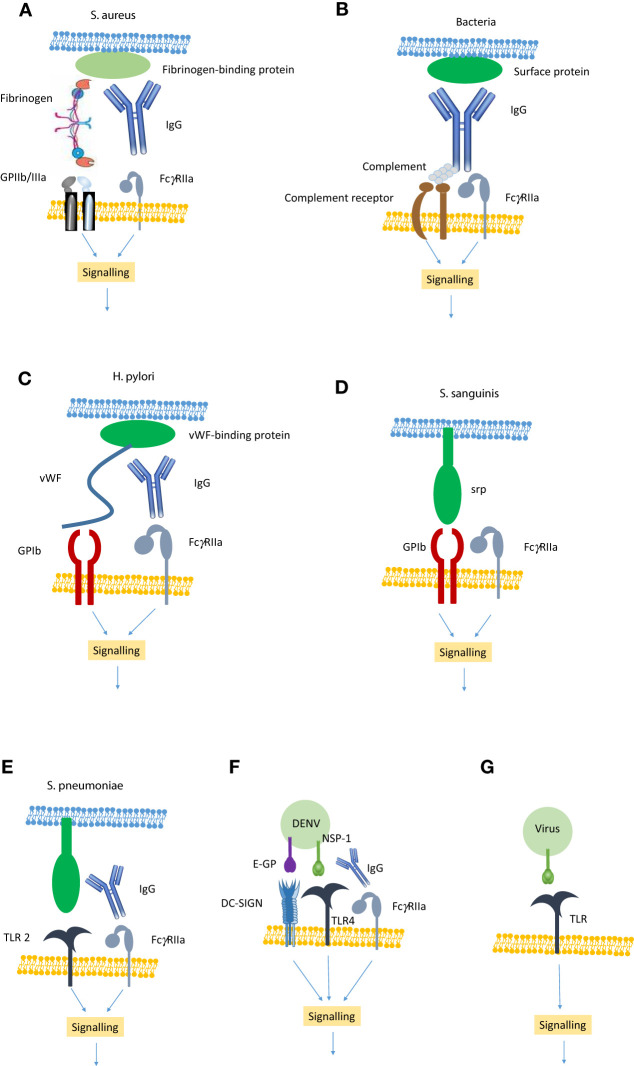
Showing the main mechanisms of pathogen-induced platelet activation. **(A)** Bacteria such as *S. aureus* express proteins that bind fibrinogen which in turn binds to GPIIb/IIIa. Simultaneously, IgG binds to the bacteria and also to FcγRIIa generating an activation signal. **(B)** Any bacteria can bind IgG which in turn leads to the assembly of complement. The IgG binds to FcγRIIa and the complement to a complement receptor to generate an activation signal. **(C)** Bacteria such as *H*. *pylori* express a protein that binds vWF, which in turn binds GPIb. IgG also binds to the bacteria and also to FcγRIIa generating an activation signal. **(D)** Bacteria such as *S. sanguinis* express proteins (e.g. serine-repeat protein; srp) that can directly bind GPIb. This generates an FcγRIIa-dependent activation signal. **(E)** Bacteria such as *S. pneumonia* express a protein that binds to Toll-like receptor (TLR) 2. In conjunction with IgG engagement of FcγRIIa they can generate an activation signal. **(F)** DENV E-glycoprotein can bind directly to DC-SIGN and non-structural protein (NSP)-1 can bind to TLR4. In conjunction with IgG binding to FcγRIIa this leads to the generation of an activation signal. **(G)** Some viruses express proteins that bind to TLR (e.g TLR 2, 4 &7) leading to a platelet activation signal.

There are two families of receptors on platelets that are involved in the interaction with pathogens – receptors involved in haemostasis such as GPIIb/IIIa and GPIb and immune receptors. The known interactions of bacteria are summarised in **Figure 1** and **Table 1**.

**Table 1:** Summary of the known pathogen proteins that interact with platelets either through a direct interaction or via a bridging protein.

Pathogen	Pathogen protein	Binding protein	Platelet receptor	Reference
**Staphylococcus aureus**	Clumping factor (Clf) A&B	Fibrinogen	GPIIb/IIIa	(36)
	Fibronectin binding protein (Fnbp)	Fibronectin	GPIIb/IIIa	(37)
	serine-aspartate repeat protein (SdrE)	-	?	(36)
	Protein A	vWf	GPIb	(38)
	IsdB	––	GPIIb/IIIa	(39)
	Peptidoglycan		TLR-2	(40)
	Extra cellular fibrinogen binding protein (Efb)*	Fibrinogen	Inhibits aggregation	(41)
	SraP	–––	?	(42)
***Staphylococcus epidermidis***	serine-aspartate repeat protein (SdrG)	Fibrinogen ––	GPIIb/IIIa GPIIb/IIIa	(43)
***Staphylococcus pseudintermedius***	SpsL	Fibrinogen	GPIIb/IIIa	(44)
***Streptococcus gordonii***	Pad A	––––	GPIIb/IIIa	(45)
	Hsa	––––	GPIb	(46)
	GspB	––	GPIb	(47)
***Streptococcus pyogenes***	M proteins	Fibrinogen	GPIIb/IIIa	(48)
***Streptococcus sanguinis***	serine-rich protein (srp)	––	GPIb	(49)
***Streptococcus oralis***	Hsa	––	GPIb	(50)
***Streptococcus pneumoniae***	?	?	TLR2	(51)
***Streptococcus agalactiae***	?	?	TLR2	(52)
	scpB-1	RGD-containing protein	GPIIb/IIIa??	(53)
	FbsA	Fibrinogen	GPIIb/IIIa	(54)
**Group G streptococci**	FOG	Fibrinogen	GPIIb/IIIa	(55)
***Helicobacter pylori***	?	vWf	GPIb	(56)
	CagL	RGD-containing protein	GPIIb/IIIa??	(57)
***Mycobacterium tuberculosis***	Peptidyl Prolyl Isomerase A	RGD-containing protein	GPIIb/IIIa??	(58)
***Borrelia burgdorferi***	BBB07	RGD-containing protein	GPIIb/IIIa??	(59)
***Leptospira interrogans***	vwa-I&II	–––	GPIb Inhibition of aggregation	(60)
**DENV**	?	–––	GPIIb/IIIa	(61)
	?	–––	GPIb	(62)
	E-glycoprotein	–––	DC-SIGN	(63)
	Non-structural protein 1	–––	TLR4	(64)
**SARS-CoV**	Spike protein	–––	DC-SIGN	(65)
**SARS-CoV-2**	Spike protein	–––	ACE2	
	Spike protein	–––	TLR4	
	Spike protein		DC-SIGN	
	Spike protein	RGD-containing protein	GPIIb/IIIa?	
**Severe fever with thrombocytopenia syndrome (SFTS) virus**	?	––––	GPVI	(66)
**Cytomegalovirus**	?	?	TLR2	(67)
** Encephalomyocarditis virus**	?	?	TLR7	(68)
**HIV**	Gp120	––––	DC-SIGN	(69)
			Clec-2	
	Tat	RGD-containing protein	GPIIb/IIIa??	(70)
**Ebola virus**	Ebola glycoprotein	–––	DC-SIGN	(71)
**Hepatitis C virus**	Envelope protein	––––	DC-SIGN	(72)
**Influenza**	Hemagglutinin Protein	––––	DC-SIGN	(73)
**All pathogens**	?	IgG	FcγRIIa	(74)
	?	Complement	Complement receptor	

Direct interactions are indicated by the absence of a binding protein (-). ? indicates that the identity of the receptor is unknown.

* indicates a secreted product.

### Glycoprotein IIb/IIIa

GPIIb/IIIa is the fibrinogen receptor that is unique to platelets. It is the dominant protein on the platelet surface and plays a critical role in platelet aggregation. It is a member of the integrin family of cell adhesion molecules and binds many proteins in an RGD-dependent manner. It also binds a fibrinogen-specific domain (γ-chain dodecapeptide). Binding of soluble ligands requires activation of GPIIb/IIIa although resting GPIIb/IIIa can bind to immobilised ligands (75).

GPIIb/IIIa mediates platelet adhesion to the sub-endothelial matrix and to other platelets and it is the fibrinogen-mediated binding to other platelets that results in thrombus formation. However, GPIIb/IIIa is also involved in platelet activation. GPIIb/IIIa binding to the NGR sequence in immobilised fibrinogen results in platelet activation and spreading (76). Furthermore, small molecule GPIIb/IIIa antagonists have been shown to activate platelets and the development of this class of drug was discontinued due to increased platelet activation leading to increased cardiovascular events (77).

GPIIb/IIIa is a key receptor for interacting with pathogens. *Staphylococcus aureus* expresses multiple fibrinogen-binding proteins including clumping factors A and B and fibronectin-binding protein. Fibrinogen-coated *S. aureus* binds to platelet GPIIb/IIIa and induces platelet activation (37). Similarly, strains of *Streptococcus pyogenes* that express fibrinogen-binding M proteins (M1, M3 and M5) can also induce platelet aggregation in a GPIIb/IIIa-dependent manner (48). Other fibrinogen-binding proteins that mediate platelet aggregation include Sdr G (*Staphylococcus epidermidis*) (43), SpsL (*Staphylococcus pseudintermedius*) (44, 55), and FOG (Group G streptococci) (55). On the other hand *S. aureus* secretes extracellular fibrinogen binding protein (Efb) which acts to inhibit platelet aggregation (41).

While GPIIb/IIIa is primarily the fibrinogen receptor and mediates that FOG interaction via the γ-chain dodecapeptide, it is also an RGD-binding integrin and is capable of binding many RGD-containing proteins. For instance GPIIb/IIIa is capable of binding fibronectin via its RGD-binding site. *S. aureus* expresses a fibronectin-binding protein (Fnbp) and binding of fibronectin to this protein mediates an interaction with GPIIb/IIIa and triggering platelet activation in a manner similar to fibrinogen-bound bacteria (37). *S. epidermidis* Sdr G (43), *S. aureus* Isd protein (78) and *S. gordonii* PadA (45) can bind directly to GPII/IIIa. DENV has been shown to directly bind to GPIIb/IIIa (61). The RGD-binding ability of GPIIb/IIIa creates another possibility. RGD-is a common peptide motif found in matrix proteins and supports cell adhesion via one of the many RGD-dependent integrins. Pathogens often express the RGD motif and use it as a virulence factor as it allows them to attach to and subsequently infect host cells. Examples of pathogens that use an RGD-containing protein to mediate adhesion to host cells include *Bordetella pertussis* (filamentous hemagglutinin) (79), *Streptococcus agalactiae* (*scpB*) (53), *Mycobacterium tuberculosis* (Peptidyl Prolyl Isomerase A) (58), *Borrelia burgdorferi* (BBB07) (59), *Helicobacter pylori* (CagL) (80). *Candida albicans* (Sap6) (81), SARS-CoV-2 (82), Coxsackievirus A9 (VP1) (83) and HIV-1 (Tat protein) (70) also express RGD-containing proteins. As GPIIb/IIIa is capable of binding many RGD-containing proteins it is likely that it can also bind RGD-containing pathogen proteins. **Figure 1A** illustrates GPIIb/IIIa-mediated platelet activation by fibrinogen-binding pathogens)

### GPIb

After GPIIb/IIIa, the most highly expressed protein on the platelet surface is GPIb which exists as a complex with GPIX and GPV. GPIb is a receptor for von Willebrand Factor (vWF) and this interaction mediates platelet adhesion to matrix-associated vWF but only under high shear conditions. This interaction leads to platelet spreading and activation (84). Pathogens also use GPIb to facilitate an interaction with platelets. One way to do this is by the expression of vWF-binding proteins on the bacteria surface. *H. pylori* binds vWF and induces platelet aggregation in a GPIb-dependent manner (56). This is unusual as high shear is not necessary for this interaction. Thus, it is likely that when bound to the surface of *H. pylori* vWF-undergoes a conformational change that allows it to interact with GPIb without the need for shear.

Pathogens also express proteins that can directly bind to GPIb. Examples of GPIb-binding proteins include serine-rich protein (srp) A on *Streptococcus sanguinis* (49) and Hsa on *Streptococcus gordonii* (46) and *Streptococcus oralis* (50). On the other hand *Leptospira interrogans* secretes a vWF-like proteins (vwa-I&II) that bind to GPIb and play a role in the haemorrhagic shock by blocking the GPIb-vWF interaction (60). Dengue virus appears to interact with GPIb although the mechanism is unknown (62). **Figures 1C, D** illustrate GPIb-dependent pathogen-induced platelet aggregation.

### Other receptors

ACE2 has been found to be expressed on platelets and mediates SARS-CoV-2 binding (85). Severe fever with thrombocytopenia syndrome (SFTS) virus (SFTSV) binds to platelet GPVI and induces platelet activation. Furthermore, SFTSV can enter platelets and replicate (66).

## Pathogen interactions with platelet immune receptors

As innate immune cells, platelets express multiple immune receptors such as FcγRIIa and Toll-Like receptors (TLRs). While these are not involved in haemostasis they do play a role in platelet activation by pathogens. Many pathogens bind to GPIIb/IIIa or GPIb which is critical in the activation of platelets, however, these interactions are insufficient to induce platelet activation. In all case platelet activation and subsequent aggregation is FcγRIIa-dependent (74).

Aside from the haemostasis resceptors that mediate the unique haemostasis functions of platelets, platelets also express multiple receptors that are usually associated with immune function. These include FcγRIIa, Toll-like receptors (TLR) and Clec-2. The immune receptors are also involved in pathogen-mediated platelet activation.

### FcγRIIa

It is clear that many pathogens can bind to, and activate, platelets by interacting with the major platelet surface receptors, however, while these interactions are necessary for platelet activaton they are not sufficient, as signalling through FcγRIIa was also essential in all cases. In haemostasis, direct activation of a platelet receptor is sufficient to induce thrombus formation. However, in immunology co-stimulation is the norm with multiple signals being required for immune cell activation.

Fc receptors are a super-family of receptors that bind the Fc portion of antibody. Each antibody class has its own Fc family (IgA/FcαR, IgG/FcγR and IgE/FcεR) that mediates immune cell activation by immune complexes. While the best known reaction is that of IgE complexes with FcεR on basophils leading to histamine release and anaphylaxis, by far the most widely expressed FcR is the FcγR family (86, 87).

FcγR is a family of receptors whose primary function is to mediate phagocytosis and thus their expression on phagocytic cells, although surprisingly, they are also expressed on platelets. There are three sub-families of FcγR – FcγRI, FcγRII and FcγRIII and these differ in their affinities for the different IgG isotypes. The most important FcγR sub-family is FcγRII and it is compsoed of FcγRIIa and FcγRIIb. Aside from cellular distribution, these 2 receptors differ in their signalling. FcγRIIa, like other FcγR signals through an immunoreceptor tyrosine-based activation motif (ITAM). ITAM is characterised by a tyrosine residue separated from a leucine or isoleucine by 2 amino acids (YxxL/I). ITAMs contain 2 of these domains separated by 6-8 amino acids. Receptor activation leads to phosphorylation of the tyrosines in ITAM by Src family kinases. These phosphtyrosines then recruit the tyrosine kinase Syk, which commences the signalling cascade. FcγRIIb, however, contains an immunoreceptor tyrosine-based inhibitory motif (ITIM) that recruits phosphatases and thus is an inhibitory receptor (88).

FcγRII all have an ITAM/ITIM domain as part of the cytoplasmic tail of the receptor. Other FcRs such as FcγRI interact with an ITAM-containing adapter protein known as FcRγ (89). FcRγ also mediates signalling by the collagen receptor GPVI on platelets (90).

FcγRIIa is the only FcR on platelets where it plays a key role as a co-stimmulatory receptor for pathogen-induced aggregation (91). Its primary function is to bind pathogen-bound IgG. Extensive work with *S. aureus* showed that while binding to GPIIb/IIIa was critical in its interaction with platelets, activation only occurs in the presence of anti-*S. aureus* IgG which engages with FcγRIIa (92). As *S. aureus* is a commensal, nearly everbody has significant titres of anti-*S. aureus* IgG. In the case of *H. pylori*, which is not a commensal, platelet activation only occurs with platelets from *H. pylori*-positive individuals (56). *Streptococcus bovis/Spreptococcus equinus* complex also induce platelet aggregation in a FcγRIIa-dependent manner (93). Peptidoglycan from *Bacillus anthracis* can also induce platleet activation in an FcγRIIa- and IgG-dependent manner (94). Viruses can also cause platelet activation in an FcγRIIa-dependent manner. DENV triggers platelet activation in an IgG and FcγRIIa-dependent manner (95). These anti-DENV antibodies arise from a prior DENV infection with a different serotype. Influenza H1N1 (96) and some Bunyaviruses such as Crimea-Congo Haemorrhagic fever (97), also induce platelet activation via FcγRIIa. Antibodies to Spike protein have been found to lead to platelet activation in COVID-19 patients that was FcγRIIa-dependent (98). Blocking FcγRIIa has been found to prevent platelet activation by COVID-19 plasma *in vitro* (99, 100). In all cases blockade of FcγRIIa with an antibody or depletion of specific IgG inhibits platelet activation, confirming the role of the IgG- FcγRIIa interaction in platelet activation.

Platelet FcγRIIa is not only involved in pathogen-induced platelet activation but it is also involved in immune thrombocytopenia (ITP), where antibodies to platelet antigens trigger the immune destruction of platelets (101). This depletion of platelets in ITP is not the just the usual immune destruction, as these antibodies trigger platelet activation in an FcγRIIa-dependent manner. This is also found in heparin-induced thrombocytopenia (HIT) where heparin binds to PF4 on the platelet surface and in some individuals this complex can become antigenic. Antibodies bind to the complex and also engage FcγRIIa leading to platelet activation and consumption which presents as a severe thrombocytopenia.

While FcγRIIa is critical in platelet activation by pathogens the same is not true of IgG. *Streptococcus sanguinis* mediates platelet activation by engaging GPIb and the aggregation is inhibited by antibodies that block FcγRIIa, however, this aggregation occurs in the absence of IgG (102). This suggests that in this context, FcγRIIa is acting as a co-receptor for GPIb. This may be explained by evidence of co-localisation of GPIb with FcγRIIa (103).

A major challenge in investigating the role of FcγRIIa in thrombosis is its restricted expression. FcγRIIa is not expressed in mice and these are the primary animal model for both thrombotic and infectious disease. Thus, pathogen-induced platelet activation in mice occurs in an FcγRIIa-independent manner and is likely to be quite different to that in humans. This is supported by evidence of differences in gene expression between mice and humans in sepsis and trauma. This species difference has clinical consequences with activated protein C (APC) being approved for the treatment of sepsis based on animal studies (31) and yet had to be withdrawn from the market due to a lack of efficacy (32).

### Toll-like receptors

TLRs are also expressed on platelet surface and these have been shown to be involved in pathogen interaction with platelets (40, 104). Most of the attention was focused on TLR2 and TLR4 as they are the most widely expressed TLRs. There are conflicting data regarding the functionality of these TLR in platelet function (105, 106). Lipopolysaccharide (LPS) is a TLR4 agonist and LPS from *E. coli* O157 fails to induce platelet aggregation even though *E. coli* O157 can induce aggregation, which suggests that TLR4 is not functional (39). The conflicting data appear to relate to the end point of any study. Thus, studies that use aggreation as the end point see no role for TLR4 while those that look at other markers of platelet activation do see a role. In contrast, the TLR2 agonist Pam3Csk4 can induce platelet aggregation (39), *Streptococcus pneumoniae* (51), *Streptococcus agalactiae* (52) and cytomegalovirus (67, 107) induce platelet aggregation in a TLR2-dependent manner. SARS-CoV-2 envelope protein has been shown to interact with TLR-2 (108). Encephalomyocarditis virus has been shown to bind to and activate platelets in a TLR7-dependent manner (68). SARS-CoV-2 has been shown to bind to TLR-4 thereby activating platelets (109). A key role for pathogen-binding to platelet TLR is to facilitate the platelet-leucocyte interaction (110, 111) and it may play a role in endocytosis of virions by platelets (112). TLR-4 has also been shown to induce platelet pyroptosis in a TLR-4-dependent manner (29). **Figures 1D, G** illustrate the role of TLRs in pathogen-induced platelet activation.

### Lectins

Lectins are a family of receptors that recognise carbohydrates but can also bind to proteins. C-type lectins are calcium-dependent lectins that act as pathogen-recognition receptors (113). C-type lectins such as DC-SIGN (Dendritic Cell-Specific Intercellular adhesion molecule-3-Grabbing Non-integrin) and CLEC (C-type lectin-like receptor) 2 are expressed on platelets. Clec-2 signalling is similar to FcγRIIa (90) although it only has a single YxxL/I domain known as a hemITAM (114). DC-SIGN binds HIV (69), DENV (63, 115, 116), Ebola virus (71), Hepatitis virus (72), and influenza H1N1 (117) and H5N1 (73), SARS-CoV (118) and SARS-CoV-2 (119, 120) and this binding is implicated in platelet activation. CLEC-2 and 5A are also important in binding to viruses (121) such as HIV (69).

### Complement receptors

Complement formation in response to an infection is an important feature of the innate immune system. Platelets can become activated in response to complement. Furthermore, complement-bound immune complexes can bind to both FcγRIIa and complement receptors gC1q-R thereby triggereing platelet aggregation (122). Once platelet-binding proteins have been removed from both *S. sanguinis* (123) and *S. aureus* (92) they can induce platelet aggregation in a complement- and FcγRIIa-dependent manner. COVID-19 is associated with increased complement formation (124, 125) and complement fragments have been found in coronary micro-thrombi of COVID-19 patients after autopsy. SARS-CoV-2 can directly activate complement formation via the alternative pathway (126) and patients who died had higher levels of anti-N antibodies that fixed complement (127). The glycosylation profile of the anti-SARS-CoV-2 IgG also appears to be important with a low fucosylated form being more pro-inflammatory (128) and more pro-thrombotic (129). The role of complement in pathogen-induced platelet activation is illustrated in **Figure 1B**.

## Interactions of secreted bacterial products with platelets

While direct interactions of bacteria with platelets are critical in the subsequent platelet activation, bacteria also secrete many products including toxins that have the potential to interact with platelets (130). *S. aureus* secretes a number of substances that have been shown to activate platelets including extracellular adherence protein Eap, chemotaxis inhibitory protein of *S. aureus* (CHIPS), formyl peptide receptor-like 1 inhibitory protein (FLIPr) and the autolysin Atl (131). *S. aureus* also secretes the prothrombin activating proteins staphylocoagulase and vWf-binding protein that can induce platelet activation through increased thrombin production (132). There is also a family of pore-forming toxins that act like the calcium ionophore (A23187) (133). These include pneumolysin (*S. pneumonia*), Streptolysin O (Group A streptococci) and α-toxin (*S. aureus*) (130). Shiga toxin is produced by some strains of *E. coli* and plays an important role in the pathogenesis of haemolytic uremic syndrome (HUS). Shiga toxin acts in conjunction with LPS to activate platelets leading to the generation of NETS (134). Staphylococcal superantigens and staphylococcal superantigen-like protein (SSL) also activate platelets. SSL5 has been shown to activate platelets by binding to GPIbα and GPVI (135). *S. aureus* toxic shock syndrome toxin-1 (TSST-1) mediates platelet activation and apoptosis, although the mechanism of TSST-1-mediated platelet activation is unknown (136). *Porphyromonas gingivalis* secretes gingipains that can mimic thrombin and activate platelets by cleaving protease-activated receptors (PAR) (137–139).

## Platelet-infected cell interactions

While *in vitro*, pathogen interactions with platelets can easily be investigated, it is much more complex *in vivo* as multiple cell types, especially endothelial cells also play a role. Both bacteria and viruses can infect endothelial cells and platelets can play a role here. Typically, resting platelets and resting endothelial cells do not interact but activated platelets will bind to activated endothelial cells. Furthermore, activated platelets will enhance endothelial cell activation and vice versa (140, 141). *S. aureus* can bind to endothelial cells using multiple virulence factors and once bound they can attract platelets (142). Endothelial cells infected with Cytomegalovirus (CMV) induce platelet adhesion and aggregation that is vWF and GPIb-dependent (143). This immunothrombosis in response to endothelial cell infection may play a role in the increase in atherosclerosis and mortality post-infection (144).

## Beyond thrombus formation

While the role of pathogen-platelet interactions in thrombus formation is well established these interactions also lead to activation of the immune system (145). The formation of neutrophil extracellular traps (NETs) – a mesh of neutrophil-derived chromatin fibres – plays an important role in trapping and killing pathogens (146). Full NET formation requires the formation of platelet-neutrophil complexes in a TLR4-dependent process (147). S100A8/A9 binding to TLR-4 induces platelet pyroptosis and these platelets are very potent at inducing NETosis, furthermore NETs release S100A8/A9 which increases platelet pyroptosis (29). Platelet-derived exosomes isolated from sepsis patients have been shown to induce NET formation (148). There is evidence that eosinophils (EET) and monocytes (MET) can also form extracellular traps (149). Platelet-eosinophil interactions are involved in EET formation (150, 151). Platelet-monocyte aggregate formation is also implicated in sepsis (152) and the have been shown to mediate killing of *Klebsiella pneumonia* (153). In severe COVID-19 (154) and Dengue (155) activated platelets activate monocytes and are implicated in disease severity.

Platelet activation by pathogens can also lead to release of granule contents that are rich in cytokines and can influence the immune response. DENV infection of platelets induces NO production and IL-1β release (27). SARS-CoV-2 (156) and DENV (157) have been shown to induce the release of multiple pro-inflammatory cytokines from platelets.

## Pathogen-induced platelet activation: clinical implications

The innate immune role of platelets in responding to infection is not restricted to bacteria as they also play an important role in responding to viruses (158–161). The ability of pathogens to activate platelets has clinical implications. Infection is associated with platelet activation, and this is part of the pathogenesis of many infectious diseases. Platelet activation is associated with two types of infection – localised infection (infective endocarditis) and systemic infection (sepsis). Evidence for platelet activation being involved in the pathogenesis of infectious disease opens the possibility of anti-platelet agents in the treatment of some infectious diseases.

One of the best characterised interactions with viruses is the DENV-platelet interaction. There are 4 serotypes of DENV. Infection with any serotype leads to a minor infection with ‘flu-like symptoms. The immune response soon clears the virus, and the patient is immune from future infection. However, if the patient is infected with a different DENV serotype there will be an immune response from the pre-existing anti-DENV antibodies, although these are not inhibitory antibodies for the new serotype. As a result, there are antibody-coated DENV virions in the circulation. These can interact with FcγRIIa on monocytes, leading to antibody-dependent enhancement (ADE) of the infectivity of DENV (95). The antibody-coated DENV virions can also bind to FcγRIIa on platelets which leads to platelet activation, DIC and Dengue haemorrhagic fever (DHF) (162, 163). **Figure 1F** illustrates DENV-induced platelet activation.

### Infective endocarditis

Infective endocarditis (IE) is due to infection of cardiac valves. This can be infection of healthy valves or compromised valves. One of the primary triggers for IE is rheumatic fever, which is an inflammatory disorder that arises from untreated streptococcal infection of the throat (164). One complication of rheumatic fever is damage to the cardiac valves which makes then susceptible to future infections. However, the use of antibiotics to manage streptococcal throat infections has greatly reduced the incidence of rheumatic fever. Another significant cause of IE is intravenous drug use which likely leads to damage to cardiac valves creating a susceptibility to infection. Finally, prosthetic cardiac valves are also risk factors for developing IE (165, 166).

While many bacteria can cause IE most cases are due to infection by staphylococci or streptococci, both of which are well known to induce platelet aggregation. As a result, when the valve becomes infected, platelets are attracted to the lesion, bind to the bacteria, and become activated. Once activated they recruit more platelets and form a thrombus. As the thrombus grows it can put a strain on the valve causing the valve to fail. The thrombus can become unstable due to the physical stress applied to it as the valve opens and closes. This can cause the thrombus to fracture and embolise which can lead to a stroke, myocardial infarction or pulmonary embolism depending on where the embolus becomes trapped (167). IE embolism has a high mortality rate.

Platelets are recruited to the infected valve to manage the infection. Once activated they secrete anti-microbial peptides to kill the bacteria and recruit immune cells. However, if these bacteria are resistant to the anti-microbial peptides they can continue to grow and, as the bacteria are surrounded by platelets, the immune cells cannot gain access to bacteria. Furthermore, by being surrounded by platelets antibiotics may not work as they also cannot gain access to the bacteria within the thrombus. Thus, if bacteria are resistant to platelet-derived anti-microbial peptides the ability to activate and recruit platelets is an effective survival mechanism.

The significant role for platelets in the development of infected thrombi suggests a role for anti-platelet agents in managing patients with IE (168, 169). However, the data have been mixed. Animal studies have shown reduced thrombus size, but clinical studies have been mixed with some showing benefit (170) and others no benefit. However, it is worth noting that these studies were small and no data on the causative agents or the role of antibiotics (169).

### Sepsis

While IE is an example of a focal infection that triggers thrombus formation, sepsis is a systemic infection and thus there is no localised thrombus formation. However, platelets still interact with the bacteria and become activated while in the circulation. These activated platelets can clump together forming thrombi that can occlude the microvasculature. This results in ischemic damage to the surrounding tissue (this can be organs such as kidney, liver, and brain). As platelet activation spreads so too does the ischemic damage which, if it becomes extensive, can lead to organ failure.

Once bacteria gain access to the circulation, they interact with platelets leading to their activation and consumption. In response to this platelet consumption, there is an increase in platelet production and thus in early-stage sepsis it is common to see an increase in platelet count. However, soon the synthetic ability of the body is overcome as the rate of platelet consumption exceeds the rate of platelet production. At this point the patient begins to develop thrombocytopenia.

The occurrence of thrombocytopenia is well established in sepsis although its occurrence may simply be an association – a secondary event to increased inflammation during sepsis- rather than a causative factor in sepsis. However, there is a clear association between the extent of thrombocytopenia and outcome in sepsis. Furthermore, it is interesting to note that many cases of culture-positive sepsis are due to infection with Staphylococci, Streptococci and *E. coli* –all of which have been shown to directly activate platelets. Thus, direct activation of platelets by bacteria is a more likely cause of thrombocytopenia than secondary activation due to increased inflammation.

The role of platelets in infection is a double-edged sword. Platelet activation by bacteria leads to the release of anti-microbial peptides (171) and also enhances the ability of monocytes to kill *Klebsiella pneumoniae (* 153 *)* and thus platelets play an important role in preventing sepsis. Thrombus formation acts to trap bacteria and prevent dissemination of the bacteria (172) although there appears to be an organ-specific effect on the ability to trap *Salmonella Typhimurium* (173). Thrombus formation occurred with *S. Typhimurium* infection of mice in a platelet Clec-2-dependent manner (174). However, specific deletion of platelet Clec-2 has been shown to enhance inflammation and organ damage in a caecal-ligation sepsis model (175). Depletion of GPVI but not Clec-2 has been shown to increase bacterial load and decrease inflammation in lungs after *K. pneumoniae* infection (176). There is also evidence that platelets can aid in dissemination of bacteria (*Streptococcus pyogenes*) (177). Thrombocytopenic mice have more severe sepsis (polymicrobial sepsis) (178) with impaired survival after *K. pneumoniae* (179) and *Streptococcus pneumoniae* (180) infection, although it lead to more severe sepsis with *S. aureus* infection (181). On the other hand, excessive platelet activation is critical to the pathogenesis of sepsis. As far back as 1981 aspirin was shown to reduce the impact of sepsis in mice (*Salmonella enteritidis*) (182, 183). It has been shown to protect mice from *S. aureus*-induced sepsis (184). Clopidogrel (185) and ticagrelor (186) were found to be protective in a polymicrobial model of sepsis although clopidogrel has also been shown to be of no benefit (187). In mouse studies of influenza-induced pneumonia the use of an anti-viral agent in conjunction with clopidogrel reduced mortality (188).

Thus, the above animal studies on sepsis have had contradictory results. Depletion of platelets prior to sepsis generally leads to worse outcome, although some studies have shown the opposite. Anti-platelet agents have been shown to be beneficial in sepsis although some studies found no effect. These conflicting studies make it difficult to understand the role of platelets in sepsis. Some of the differences can be due to the inducing agent – endotoxin versus live pathogen. Even with live pathogen there can be differences in whether a Gram-negative or Gram-positive pathogen is used, or whether it is a polymicrobial infection or a single pathogen. However, it does appear that the presence of platelets is important in protecting against sepsis due to their role in innate immunity. Sepsis only arises if the pathogen is resistant to the platelet anti-microbial peptides, or the dose of pathogen is so high that it overcomes the innate immune system. So not surprisingly platelet depletion can worsen sepsis outcomes possibly by inducing sepsis in animals that would normally clear the pathogen. However, if sepsis is established, the innate immune role of platelets has failed. At this point the platelet becomes part of the problem rather than the solution to the problem and thus, platelet inhibition is likely to be beneficial.

If direct platelet activation by bacteria plays a critical role in the development of multi-organ failure in sepsis, then anti-platelet agents should improve outcome in sepsis. While some clinical studies have found no evidence for reduced incidence of sepsis or subsequent mortality (189, 190) with the use of aspirin, other studies have shown benefit. Aspirin use prior to admission to ICU has been shown to reduce sepsis mortality (191, 192) and mortality from pneumococcal pneumonia (193). Lavie and co-workers (194) and Du and co-workers (195) used propensity matching to show that aspirin use reduced mortality in sepsis (hazard ratio approximately 0.7). Meta-analysis (196) have shown that prior aspirin and/or clopidogrel use was associated with a reduction in sepsis mortality of around 10%. The interesting thing about these studies is that patients with prior aspirin use are typically patients post myocardial infarction. Thus, patients with significant underlying health issues who are on aspirin do better than those with no underlying conditions.

The potential benefits of aspirin are not just restricted to preventing sepsis. A serious infection that requires hospitalisation (not sepsis) is associated with a significant increase in major cardiovascular events (MACE) - hazard ratio for MACE for the first month post-infection was 7.87 and for the following 19-years it was 1.41 (197). The increase in MACE was also seen in Dengue fever where the incidence rate ratio (IRR) of MACE in the 7-days post DENV infection was 17.9, post-influenza 15.76 and in the control group 0.91 (198). Risk factors for MACE post-infection were shown to be evidence of organ damage, atrial fibrillation and at least 2 risk factors of MACE (199). Similarly, the odds ratio for MACE in COVID-19 patients was 6 (200). These results are not surprising as MACE is due to platelet activation and subsequent thrombus formation. The last thing that a patient at risk of an MI needs is significant increase in platelet activation such as that which occurs when pathogens interact with platelets. Thus, the use of aspirin in patients with serious infection, especially in those with CVD risk factors has a role in preventing MACE following the infection.

One of the reasons for the diversity in outcomes may be due to timing of aspirin use. Just as in the animal studies, the presence of healthy, fully functional platelets is necessary to fight infection and thus instances of infection that may be resolved with the aid of platelets may progress to sepsis. The ideal time to administer aspirin would be when there is evidence that the infection has become established, i.e., at the point where there is evidence that the platelets have failed to contain the infection, but before there is significant thrombocytopenia as it is difficult to preserve platelet function if there are no platelets remaining.

However, there is a problem with using conventional anti-platelet agents in sepsis. As thrombocytopenia progresses so too does the bleeding risk. Anti-platelet agents also create a bleeding risk. Thus, the use of anti-platelet agents may preserve platelet number but increase the bleeding risk by impairing platelet function. An alternative strategy is to prevent the pathogen from activating the platelets. As FcγRIIa is the most significant platelet receptor mediating pathogen-induced platelet activation it would be the ideal drug target. It would prevent pathogen-induced platelet activation without any impact on platelet function. There is evidence to support this concept from studies using IVIg which is known to act by inhibiting FcγRIIa (201). High dose IVIg, if given early, improves outcome in severe COVID-19 infection (202). A network meta-analysis found that IVIg reduced mortality of sepsis in adults (odds ratio = 0.61) with an optimal dose of 1.5-2 g/kg (203). In contrast the use of IVIg in neonatal sepsis has shown no evidence of benefit (204, 205). However, it is worth noting that the doses used in the neonatal studies (500 mg/kg) are much lower than those found to be optimal in adults (1.5-2 g/kg). The American Heart Association guidelines (206) recommend 2 g/kg IVIg for children with Kawasaki Disease, noting that benefit is dose-dependent, which suggests there was a significant under-dosing in the neonatal sepsis studies. While IVIg is an FcγRIIa antagonist it has low affinity for the receptor and thus high concentrations are required to get significant inhibition. Kawasaki disease is believed to be triggered by an infection, although the identities of the causative agents is unknown, and it is treated with a combination of aspirin and IVIg – a strategy that would protect platelets from direct activation of platelets by a pathogen (206). IVIg has been shown to be effective in Crimea-Congo Haemorrhagic fever (97), Influenza A (H1N1) (207) and COVID-19 (202, 208). A potential solution to the low affinity of IVIg is to discover high affinity small molecules. Small molecules have been discovered that have been shown to be effective in animal models of immune complex disease (209).

A key step in NETosis is the release of DNA which plays a role in trapping platelets (146). This has led to investigations on the use of DNase in sepsis although with mixed results that may be due to timing of DNase therapy (210–212). However, as platelet activation is critical in NETosis (147), anti-platelet agents may be more effective in regulating extracellular DNA.

## Pandemic preparedness

COVID-19 has made the world realise that we are very vulnerable to a potential pandemic. While this may come from a known pathogen, the real threat comes from a novel pathogen – possible one that recently jumped species. While there is interest in discovering a pan-anti-viral inhibitor this seems unlikely as there are so many different viruses and existing agents are specific to a small number of viruses. An alternative approach is to target the host – all pathogens must interact with the host to cause illness and there is a much smaller repertoire of targets in the host.

With many pandemics, both bacterial and viral, death follows from disseminated intravascular coagulation and multi-organ failure. Covid-19 causes multi-organ failure and is a viral sepsis (65). Influenza A, both seasonal influenza and pandemic variants (H1N1 (1918 & 2009), H2N2 (1957) and H3N2 (1968)) can lead to multi-organ failure (213). Other viruses with the potential to become pandemics are the viral haemorrhagic fevers, including Marburg, Ebola and Dengue all of which cause DIC and multi-organ failure (214–216).

Just as anti-platelet agents have the potential to prevent DIC in bacterial sepsis and reduce mortality, their use in viral sepsis has the potential for similar benefit. The real advantage is that knowledge of the pathogen is not necessary to provide benefit.

## Conclusions

Sepsis – both bacterial and viral – is associated with disseminated intravascular coagulation (DIC). This coagulopathy is characterised by extensive platelet activation that results in thrombus formation in the microvasculature leading to multi-organ failure. Ultimately it is this uncontrolled platelet activation that is the cause of mortality in sepsis. Preliminary data supports the idea of using anti-platelet agents to treat sepsis. These agents do not cure sepsis; however, they do stabilise the patient preventing progression to DIC. This buys time for the clinician to identify the cause of the sepsis and to select the appropriate antibiotic. Furthermore, it may prove beneficial in the 40% of sepsis cases that are culture-negative (217) where antibiotics have no role.

As anti-platelet agents create a risk of bleeding an alternative strategy is to discover agents that inhibit pathogen interaction with platelets. The two key receptors are FcγRIIa and DC-SIGN and inhibitors of these receptors have the potential to prevent pathogen-induced platelet activation without an increase in bleeding risk.

## Author contributions

The author confirms being the sole contributor of this work and has approved it for publication.

## References

[1] Delvaeye M Conway EM. Coagulation and innate immune responses: can we view them separately? Blood (2009) 114:2367–74. doi: 10.1182/blood-2009-05-199208 19584396

[2] Engelmann B Massberg S. Thrombosis as an intravascular effector of innate immunity. Nat Rev Immunol (2013) 13:34–45. doi: 10.1038/nri3345 23222502

[3] Sharma S Tyagi T Antoniak S. Platelet in thrombo-inflammation: unraveling new therapeutic targets. Front Immunol (2022) 13:1039843. doi: 10.3389/fimmu.2022.1039843 36451834 PMC9702553

[4] Stark K Massberg S. Interplay between inflammation and thrombosis in cardiovascular pathology. Nat Rev Cardiol (2021) 18:666–82. doi: 10.1038/s41569-021-00552-1 PMC8100938 33958774

[5] Zaid Y Merhi Y. Implication of platelets in immuno-thrombosis and thrombo-inflammation. Front Cardiovasc Med (2022) 9:863846. doi: 10.3389/fcvm.2022.863846 35402556 PMC8990903

[6] Estevez B Du X. New concepts and mechanisms of platelet activation signaling. Physiology (2017) 32:162–77. doi: 10.1152/physiol.00020.2016 PMC5337829 28228483

[7] Woulfe DS. Platelet G protein-coupled receptors in hemostasis and thrombosis. J Thromb Haemost (2005) 3:2193–200. doi: 10.1111/j.1538-7836.2005.01338.x 16194198

[8] Cremer SE Catalfamo JL Goggs R Seemann SE Kristensen AT Szklanna PB. The canine activated platelet secretome (CAPS): a translational model of thrombin-evoked platelet activation response. Res Pract Thromb Haemostasis (2021) 5:55–68. doi: 10.1002/rth2.12450 33537530 PMC7845059

[9] Verhoef JJF Barendrecht AD Nickel KF Dijkxhoorn K Kenne E Labberton L. Polyphosphate nanoparticles on the platelet surface trigger contact system activation. Blood (2017) 129:1707–17. doi: 10.1182/blood-2016-08-734988 PMC5364341 28049643

[10] Franchini M Veneri D Lippi G. Thrombocytopenia and infections. Expert Rev Hematol (2017) 10:99–106. doi: 10.1080/17474086.2017.1271319 27936979

[11] Vandijck DM Blot SI De Waele JJ Hoste EA Vandewoude KH Decruyenaere JM. Thrombocytopenia and outcome in critically ill patients with bloodstream infection. Heart Lung (2010) 39:21–6. doi: 10.1016/j.hrtlng.2009.07.005 20109983

[12] Johansson D Rasmussen M Inghammar M. Thrombocytopenia in bacteraemia and association with bacterial species. Epidemiol Infect (2018) 146(10):1312–7. doi: 10.1017/S0950268818001206 PMC9134296 29759089

[13] Kim SM Kim SI Yu G Kim JS Hong SI Kim WY. Hypercoagulability in septic shock patients with thrombocytopenia. J Intensive Care Med (2022)37(6):721–7. doi: 10.1177/08850666211024188 34105409

[14] Raadsen M Du Toit J Langerak T Van Bussel B Van Gorp E Goeijenbier M. Thrombocytopenia in virus infections. J Clin Med (2021) 10(4):877. doi: 10.3390/jcm10040877 33672766 PMC7924611

[15] Ho T-S Wang S-M Lin Y-S Liu C-C. Clinical and laboratory predictive markers for acute dengue infection. J Biomed Sci (2013) 20:75. doi: 10.1186/1423-0127-20-75 24138072 PMC4015130

[16] Zhang J Huang X Ding D Tao Z. Platelet-driven coagulopathy in COVID-19 patients: in comparison to seasonal influenza cases. Exp Hematol Oncol (2021) 10:34. doi: 10.1186/s40164-021-00228-z 34059141 PMC8165133

[17] López R Vial C Graf J Calvo M Ferrés M Mertz G. Platelet count in patients with mild disease at admission is associated with progression to severe hantavirus cardiopulmonary syndrome. Viruses (2019) 11(8):693. doi: 10.3390/v11080693 31366116 PMC6724000

[18] Jiang Q Mao R Wu J Chang L Zhu H Zhang G. Platelet activation during chronic hepatitis b infection exacerbates liver inflammation and promotes fibrosis. J Med Virol (2020) 92:3319–26. doi: 10.1002/jmv.25641 31769518

[19] Páez-Guillán EM Campos-Franco J Alende R Gonzalez-Quintela A. Hematological abnormalities beyond lymphocytosis during infectious mononucleosis: Epstein-Barr virus-induced thrombocytopenia. Mediterr J Hematol Infect Dis (2023) 15(1):e2023023. doi: 10.4084/mjhid.2023.023 36908863 PMC10000900

[20] Campbell RA Schwertz H Hottz ED Rowley JW Manne BK Washington AV. Human megakaryocytes possess intrinsic antiviral immunity through regulated induction of IFITM3. Blood (2019) 133:2013–26. doi: 10.1182/blood-2018-09-873984 PMC6509546 30723081

[21] Vogt MB Lahon A Arya RP Spencer Clinton JL Rico-Hesse R. Dengue viruses infect human megakaryocytes, with probable clinical consequences. PloS Negl Trop Dis (2019) 13:e0007837. doi: 10.1371/journal.pntd.0007837 31765380 PMC6901235

[22] Roncati L Ligabue G Nasillo V Lusenti B Gennari W Fabbiani L. A proof of evidence supporting abnormal immunothrombosis in severe COVID-19: naked megakaryocyte nuclei increase in the bone marrow and lungs of critically ill patients. Platelets (2020) 31:1085–9. doi: 10.1080/09537104.2020.1810224 32857624

[23] Nishimura S Nagasaki M Kunishima S Sawaguchi A Sakata A Sakaguchi H. IL-1α induces thrombopoiesis through megakaryocyte rupture in response to acute platelet needs. J Cell Biol (2015) 209:453–66. doi: 10.1083/jcb.201410052 PMC4427781 25963822

[24] Machlus KR Johnson KE Kulenthirarajan R Forward JA Tippy MD Soussou TS. CCL5 derived from platelets increases megakaryocyte proplatelet formation. Blood (2016) 127:921–6. doi: 10.1182/blood-2015-05-644583 PMC4760093 26647394

[25] Adelborg K Larsen JB Hvas AM. Disseminated intravascular coagulation: epidemiology, biomarkers, and management. Br J Haematol (2021) 192:803–18. doi: 10.1111/bjh.17172 33555051

[26] Lumadue JA Lanzkron SM Kennedy SD Kuhl DT Kickler TS. Cytokine induction of platelet activation. Am J Clin Pathol (1996) 106:795–8. doi: 10.1093/ajcp/106.6.795 8980357

[27] Pinheiro MBM Rozini SV Quirino-Teixeira AC Barbosa-Lima G Lopes JF Sacramento CQ. Dengue induces iNOS expression and nitric oxide synthesis in platelets through IL-1R. Front Immunol (2022) 13:1029213. doi: 10.3389/fimmu.2022.1029213 36569864 PMC9767985

[28] Hamilos M Petousis S Parthenakis F. Interaction between platelets and endothelium: from pathophysiology to new therapeutic options. Cardiovasc Diagn Ther (2018) 8:568–80. doi: 10.21037/cdt.2018.07.01 PMC6232347 30498682

[29] Su M Chen C Li S Li M Zeng Z Zhang Y. Gasdermin d-dependent platelet pyroptosis exacerbates NET formation and inflammation in severe sepsis. Nat Cardiovasc Res (2022) 1:732–47. doi: 10.1038/s44161-022-00108-7 PMC9362711 35967457

[30] Su Y Zhang T Qiao R. Pyroptosis in platelets: thrombocytopenia and inflammation. J Clin Lab Anal (2023) 37:e24852. doi: 10.1002/jcla.24852 36852778 PMC10020847

[31] Bernard GR Vincent JL Laterre PF Larosa SP Dhainaut JF Lopez-Rodriguez A. Efficacy and safety of recombinant human activated protein c for severe sepsis. N Engl J Med (2001) 344:699–709. doi: 10.1056/NEJM200103083441001 11236773

[32] Martí-Carvajal Arturo J Solà I Gluud C Lathyris D Cardona Andrés F. Human recombinant protein c for severe sepsis and septic shock in adult and paediatric patients. Cochrane Database Systematic Rev (2012). doi: 10.1002/14651858.CD004388.pub6/abstract PMC6464614 23235609

[33] Yatabe T Inoue S Sakamoto S Sumi Y Nishida O Hayashida K. The anticoagulant treatment for sepsis induced disseminated intravascular coagulation; network meta-analysis. Thromb Res (2018) 171:136–42. doi: 10.1016/j.thromres.2018.10.007 30312798

[34] Goligher EC Lawler PR Jensen TP Talisa V Berry LR Lorenzi E. Heterogeneous treatment effects of therapeutic-dose heparin in patients hospitalized for COVID-19. JAMA (2023) 329(13):1066–77. doi: 10.1001/jama.2023.3651 PMC10031504 36942550

[35] Martinod K Deppermann C. Immunothrombosis and thromboinflammation in host defense and disease. Platelets (2021) 32:314–24. doi: 10.1080/09537104.2020.1817360 32896192

[36] O'brien L Kerrigan SW Kaw G Hogan M Penades J Litt D. Multiple mechanisms for the activation of human platelet aggregation by *Staphylococcus aureus*: roles for the clumping factors ClfA and ClfB, the serine-aspartate repeat protein SdrE and protein a. Mol Microbiol (2002) 44:1033–44. doi: 10.1046/j.1365-2958.2002.02935.x 12010496

[37] Fitzgerald JR Loughman A Keane F Brennan M Knobel M Higgins J. Fibronectin-binding proteins of *Staphylococcus aureus* mediate activation of human platelets via fibrinogen and fibronectin bridges to integrin GPIIb/IIIa and IgG binding to the FcgRIIa receptor. Mol Microbiol (2006) 59:212–30. doi: 10.1111/j.1365-2958.2005.04922.x 16359330

[38] O'seaghdha M Van Schooten CJ Kerrigan SW Emsley J Silverman GJ Cox D. *Staphylococcus aureus* protein a binding to von willebrand factor A1 domain is mediated by conserved IgG binding regions. FEBS J (2006) 273:4831–41. doi: 10.1111/j.1742-4658.2006.05482.x 16999823

[39] Moriarty RD Cox A Mccall M Smith SG Cox D. Escherichia coli induces platelet aggregation in an FcgammaRIIa-dependent manner. J Thromb Haemost (2016) 14:797–806. doi: 10.1111/jth.13226 26669970

[40] Ebermeyer T Cognasse F Berthelot P Mismetti P Garraud O Hamzeh-Cognasse H. Platelet innate immune receptors and TLRs: a double-edged sword. Int J Mol Sci (2021) 22:7894. doi: 10.3390/ijms22157894 34360659 PMC8347377

[41] Shannon O Flock JI. Extracellular fibrinogen binding protein, efb, from staphylococcus aureus binds to platelets and inhibits platelet aggregation. Thromb Haemost (2004) 91:779–89. doi: 10.1160/TH03-05-0287 15045140

[42] Siboo IR Chambers HF Sullam PM. Role of SraP, a serine-rich surface protein of *Staphylococcus aureus*, in binding to human platelets. Infect Immun (2005) 73:2273–80. doi: 10.1128/IAI.73.4.2273-2280.2005 PMC1087419 15784571

[43] Brennan M Loughman A Devocelle M Arasu S Chubb A Foster T. Elucidating the role of *Staphylococcus epidermidis* serine-aspartate repeat protein G in platelet activation. J Thromb Haemost (2009) 7:1364–72. doi: 10.1111/j.1538-7836.2009.03495.x 19486275

[44] Pickering AC Vitry P Prystopiuk V Garcia B Höök M Schoenebeck J. Host-specialized fibrinogen-binding by a bacterial surface protein promotes biofilm formation and innate immune evasion. PloS Pathog (2019) 15:e1007816. doi: 10.1371/journal.ppat.1007816 31216354 PMC6602291

[45] Keane C Petersen HJ Tilley DO Haworth J Cox D Jenkinson HF. Multiple sites on streptococcus gordonii surface protein PadA bind to platelet GPIIbIIIa. Thromb Haemost (2013) 110:1278–87. doi: 10.1160/TH13-07-0580 PMC4037785 24136582

[46] Kerrigan SW Jakubovics NS Keane C Maguire P Wynne K Jenkinson HF. Role of *Streptococcus gordonii* surface proteins SspA/SspB and hsa in platelet function. Infect Immun (2007) 75:5740–7. doi: 10.1128/IAI.00909-07 PMC2168320 17893126

[47] Jakubovics NS Kerrigan SW Nobbs AH Stromberg N Van Dolleweerd CJ Cox DM. Functions of cell surface-anchored antigen I/II family and hsa polypeptides in interactions of *Streptococcus gordonii* with host receptors. Infect Immun (2005) 73:6629–38. doi: 10.1128/IAI.73.10.6629-6638.2005 PMC1230909 16177339

[48] Shannon O Hertzen E Norrby-Teglund A Morgelin M Sjobring U Bjorck L. Severe streptococcal infection is associated with m protein-induced platelet activation and thrombus formation. Mol Microbiol (2007) 65:1147–57. doi: 10.1111/j.1365-2958.2007.05841.x 17662041

[49] Plummer C Wu H Kerrigan SW Meade G Cox D Douglas CW. A serine-rich glycoprotein of *Streptococcus sanguis* mediates adhesion to platelets via GPIb. Br J Haematol (2005) 129:101–9. doi: 10.1111/j.1365-2141.2005.05421.x 15801962

[50] Tilley DO Arman M Smolenski A Cox D O'donnell JS Douglas CWI. Glycoprotein ibα and FcγRIIa play key roles in platelet activation by the colonizing bacterium, streptococcus oralis. J Thromb Haemost (2013) 11:941–50. doi: 10.1111/jth.12175 23413961

[51] Keane C Tilley D Cunningham A Smolenski A Kadioglu A Cox D. Invasive *Streptococcus pneumoniae* trigger platelet activation via toll-like receptor 2. J Thrombos Haemost (2010) 8:2757–65. doi: 10.1111/j.1538-7836.2010.04093.x 20946179

[52] Xiaoyan L Hongyun L Xianming L Ping Z Yanmin G Shuangfeng X. Strains of group b streptococci from septic patients induce platelet activation via toll-like receptor 2. Clin Exp Pharmacol Physiol (2017) 44:335–43. doi: 10.1111/1440-1681.12707 27885699

[53] Tsai IA Su Y Wang YH Chu C. Alterations in genes rib, scpB and pilus island decrease the prevalence of predominant serotype V, not III and VI, of streptococcus agalactiae from 2008 to 2012. Pathogens (2022) 11(10):1145. doi: 10.3390/pathogens11101145 36297202 PMC9611264

[54] Pietrocola G Schubert A Visai L Torti M Fitzgerald JR Foster TJ. FbsA, a fibrinogen-binding protein from streptococcus agalactiae, mediates platelet aggregation. Blood (2005) 105:1052–9. doi: 10.1182/blood-2004-06-2149 15383464

[55] Svensson L Frick I-M Shannon O. Group G streptococci mediate fibrinogen-dependent platelet aggregation leading to transient entrapment in platelet aggregates. Microbiology (2015). doi: 10.1099/mic.0.000203 26511072

[56] Byrne MF Kerrigan SW Corcoran PA Atherton JC Murray FE Fitzgerald DJ. *Helicobacter pylori* binds von willebrand factor and interacts with GPIb to induce platelet aggregation. Gastroenterology (2003) 124:1846–54. doi: 10.1016/S0016-5085(03)00397-4 12806618

[57] Tegtmeyer N Hartig R Delahay RM Rohde M Brandt S Conradi J. A small fibronectin-mimicking protein from bacteria induces cell spreading and focal adhesion formation. J Biol Chem (2010) 285:23515–26. doi: 10.1074/jbc.M109.096214 PMC2906342 20507990

[58] Dubey N Khan MZ Kumar S Sharma A Das L Bhaduri A. Mycobacterium tuberculosis peptidyl prolyl isomerase a interacts with host integrin receptor to exacerbate disease progression. J Infect Dis (2021) 224:1383–93. doi: 10.1093/infdis/jiab081 33580239

[59] Hahn B Anderson P Lu Z Danner R Zhou Z Hyun N. BBB07 contributes to, but is not essential for, borrelia burgdorferi infection in mice. Microbiol (Reading) (2020) 166:988–94. doi: 10.1099/mic.0.000972 PMC7660918 32936070

[60] Fang JQ Imran M Hu WL Ojcius DM Li Y Ge YM. vWA proteins of leptospira interrogans induce hemorrhage in leptospirosis by competitive inhibition of vWF/GPIb-mediated platelet aggregation. EBioMedicine (2018) 37:428–41. doi: 10.1016/j.ebiom.2018.10.033 PMC6284457 30337247

[61] Nattapol A Yaowalak U-P Panthipa S Chanchao L Kovit P Surapol I. Dengue virus and its relation to human glycoprotein IIb/IIIa revealed by fluorescence microscopy and flow cytometry. Viral Immunol (2017) 30:654–61. doi: 10.1089/vim.2017.0090 28945165

[62] Attatippaholkun N Kosaisawe N Yaowalak UP Supraditaporn P Lorthongpanich C Pattanapanyasat K. Selective tropism of dengue virus for human glycoprotein ib. Sci Rep (2018) 8:2688. doi: 10.1038/s41598-018-20914-z 29426910 PMC5807543

[63] Simon AY Sutherland MR Pryzdial ELG. Dengue virus binding and replication by platelets. Blood (2015) 126:378–85. doi: 10.1182/blood-2014-09-598029 PMC4826145 25943787

[64] García-Larragoiti N Kim YC López-Camacho C Cano-Méndez A López-Castaneda S Hernández-Hernández D. Platelet activation and aggregation response to dengue virus nonstructural protein 1 and domains. J Thromb Haemost (2021) 19:2572–82. doi: 10.1111/jth.15431 34160117

[65] Cox D. Targeting SARS-CoV-2-Platelet interactions in COVID-19 and vaccine-related thrombosis. Front Pharmacol (2021) 12. doi: 10.3389/fphar.2021.708665 PMC8287727 34290613

[66] Fang L Yu S Tian X Fu W Su L Chen Z. Severe fever with thrombocytopenia syndrome virus replicates in platelets and enhances platelet activation. J Thromb Haemost (2023). doi: 10.1016/j.jtha.2023.02.006 36792011

[67] Assinger A Kral JB Yaiw KC Schrottmaier WC Kurzejamska E Wang Y. Human cytomegalovirus–platelet interaction triggers toll-like receptor 2–dependent proinflammatory and proangiogenic responses. Arterioscler Thromb Vasc Biol (2014) 34:801–9. doi: 10.1161/ATVBAHA.114.303287 24558109

[68] Koupenova M Vitseva O Mackay CR Beaulieu LM Benjamin EJ Mick E. Platelet-TLR7 mediates host survival and platelet count during viral infection in the absence of platelet-dependent thrombosis. Blood (2014) 124:791–802. doi: 10.1182/blood-2013-11-536003 24755410 PMC4118487

[69] Chaipan C Soilleux E Simpson P Hofmann H Gramberg T Marzi A. DC-SIGN and CLEC-2 mediate human immunodeficiency virus type 1 capture by platelets. J Virol (2006) 80:8591–8960. doi: 10.1128/JVI.00136-06 PMC1563896 16940507

[70] Cafaro A Barillari G Moretti S Palladino C Tripiciano A Falchi M. HIV-1 tat protein enters dysfunctional endothelial cells via integrins and renders them permissive to virus replication. Int J Mol Sci (2020) 22(1):317. doi: 10.3390/ijms22010317 33396807 PMC7796023

[71] Alvarez CP Lasala F Carrillo J Muniz O Corbi AL Delgado R. C-type lectins DC-SIGN and l-SIGN mediate cellular entry by Ebola virus in cis and in trans. J Virol (2002) 76:6841–4. doi: 10.1128/JVI.76.13.6841-6844.2002 PMC136246 12050398

[72] Pöhlmann S Zhang J Baribaud F Chen Z Leslie GJ Lin G. Hepatitis c virus glycoproteins interact with DC-SIGN and DC-SIGNR. J Virol (2003) 77:4070–80. doi: 10.1128/JVI.77.7.4070-4080.2003 PMC150620 12634366

[73] Yang Z-S Huang S-W Wang W-H Lin C-Y Wang C-F Urbina AN. Identification of important n-linked glycosylation sites in the hemagglutinin protein and their functional impact on DC-SIGN mediated avian influenza H5N1 infection. Int J Mol Sci (2021) 22:743. doi: 10.3390/ijms22020743 33451024 PMC7828482

[74] Fitzgerald JR Foster TJ Cox D. The interaction of bacterial pathogens with platelets. Nat Rev Microbiol (2006) 4:445–57. doi: 10.1038/nrmicro1425 16710325

[75] Coller BS. αIIbβ3: structure and function. J Thromb Haemost (2015) 13(Suppl 1):S17–25. doi: 10.1111/jth.12915 PMC4888797 26149019

[76] Moriarty R Mcmanus CA Lambert M Tilley T Devocelle M Brennan M. A novel role for the fibrinogen asn-Gly-Arg (NGR) motif in platelet function. Thromb Haemost (2015) 113:290–304. doi: 10.1160/TH14-04-0366 25413489

[77] Cox D Brennan M Moran N. Integrins as therapeutic targets: lessons and opportunities. Nat Rev Drug Discov (2010) 9:804–20. doi: 10.1038/nrd3266 20885411

[78] Miajlovic H Zapotoczna M Geoghegan JA Kerrigan SW Speziale P Foster TJ. Direct interaction of iron-regulated surface determinant IsdB of staphylococcus aureus with the GPIIb/IIIa receptor on platelets. Microbiology (2010) 156:920–8. doi: 10.1099/mic.0.036673-0 20007649

[79] Relman DA Domenighini M Tuomanen E Rappuoli R Falkow S. Filamentous hemagglutinin of bordetella pertussis: nucleotide sequence and crucial role in adherence. Proc Natl Acad Sci USA (1989) 86:2637–41. doi: 10.1073/pnas.86.8.2637 PMC286972 2539596

[80] Yadegar A Mohabati Mobarez A Zali MR. Genetic diversity and amino acid sequence polymorphism in helicobacter pylori CagL hypervariable motif and its association with virulence markers and gastroduodenal diseases. Cancer Med (2019) 8:1619–32. doi: 10.1002/cam4.1941 PMC6488209 30873747

[81] Kumar R Rojas IG Edgerton M. Candida albicans Sap6 initiates oral mucosal inflammation via the protease activated receptor PAR2. Front Immunol (2022) 13:912748. doi: 10.3389/fimmu.2022.912748 35844627 PMC9277060

[82] Robles JP Zamora M Adan-Castro E Siqueiros-Marquez L Martinez de la Escalera G Clapp C. The spike protein of SARS-CoV-2 induces endothelial inflammation through integrin α5β1 and NF-κB signaling. J Biol Chem (2022) 298:101695. doi: 10.1016/j.jbc.2022.101695 35143839 PMC8820157

[83] Zhao H Wang J Chen J Huang R Zhang Y Xiao J. Molecular epidemiology and evolution of coxsackievirus A9. Viruses (2022) 14(4):822. doi: 10.3390/v14040822 35458552 PMC9024771

[84] Bendas G Schlesinger M. The GPIb-IX complex on platelets: insight into its novel physiological functions affecting immune surveillance, hepatic thrombopoietin generation, platelet clearance and its relevance for cancer development and metastasis. Exp Hematol Oncol (2022) 11:19. doi: 10.1186/s40164-022-00273-2 35366951 PMC8976409

[85] Zhang S Liu Y Wang X Yang L Li H Wang Y. SARS-CoV-2 binds platelet ACE2 to enhance thrombosis in COVID-19. J Hematol Oncol (2020) 13(1):120. doi: 10.1186/s13045-020-00954-7 32887634 PMC7471641

[86] Hogarth PM Pietersz GA. Fc receptor-targeted therapies for the treatment of inflammation, cancer and beyond. Nat Rev Drug Discov (2012) 11:311–31. doi: 10.1038/nrd2909 22460124

[87] Chalayer E Gramont B Zekre F Goguyer-Deschaumes R Waeckel L Grange L. Fc receptors gone wrong: a comprehensive review of their roles in autoimmune and inflammatory diseases. Autoimmun Rev (2022) 21:103016. doi: 10.1016/j.autrev.2021.103016 34915182

[88] Coxon CH Geer MJ Senis YA. ITIM receptors: more than just inhibitors of platelet activation. Blood (2017) 129:3407–18. doi: 10.1182/blood-2016-12-720185 PMC5562394 28465343

[89] Hamdan TA Lang PA Lang KS. The diverse functions of the ubiquitous fcγ receptors and their unique constituent, FcRγ subunit. Pathogens (2020) 9(2):140. doi: 10.3390/pathogens9020140 32093173 PMC7168688

[90] Rayes J Watson SP Nieswandt B. Functional significance of the platelet immune receptors GPVI and CLEC-2. J Clin Invest (2019) 129:12–23. doi: 10.1172/JCI122955 30601137 PMC6307936

[91] Patel P Michael JV Naik UP Mckenzie SE. Platelet FcγRIIA in immunity and thrombosis: adaptive immunothrombosis. J Thromb Haemost (2021) 19:1149–60. doi: 10.1111/jth.15265 33587783

[92] Loughman A Fitzgerald JR Brennan MP Higgins J Downer R Cox D. Roles for fibrinogen, immunoglobulin and complement in platelet activation promoted by *Staphylococcus aureus* clumping factor a. Mol Microbiol (2005) 57:804–18. doi: 10.1111/j.1365-2958.2005.04731.x 16045623

[93] Pernow G Shannon O Öberg J Nilson B Rasmussen M. Platelet activation and aggregation induced by streptococcus bovis/Streptococcus equinus complex. Microbiol Spectr (2022) 10:e0186122. doi: 10.1128/spectrum.01861-22 36374116 PMC9769897

[94] Sun D Popescu NI Raisley B Keshari RS Dale GL Lupu F. Bacillus anthracis peptidoglycan activates human platelets through FcγRII and complement. Blood (2013) 122:571–9. doi: 10.1182/blood-2013-02-486613 PMC3724192 23733338

[95] Wang S He R Patarapotikul J Innis BL Anderson R. Antibody-enhanced binding of dengue-2 virus to human platelets. Virology (1995) 213:254–7. doi: 10.1006/viro.1995.1567 7483271

[96] Boilard E Paré G Rousseau M Cloutier N Dubuc I Lévesque T. Influenza virus H1N1 activates platelets through FcγRIIA signaling and thrombin generation. Blood (2014) 123:2854–63. doi: 10.1182/blood-2013-07-515536 24665136

[97] Erduran E Bahadir A Palanci N Gedik Y. The treatment of crimean-congo hemorrhagic fever with high-dose methylprednisolone, intravenous immunoglobulin, and fresh frozen plasma. J Pediatr Hematol Oncol (2013) 35:e19–24. doi: 10.1097/MPH.0b013e3182706444 23018575

[98] Nazy I Sachs UJ Arnold DM Mckenzie SE Choi P Althaus K. Recommendations for the clinical and laboratory diagnosis of vaccine-induced immune thrombotic thrombocytopenia (VITT) for SARS-CoV-2 infections: communication from the ISTH SSC subcommittee on platelet immunology. J Thromb Haemost (2021) 19(6):1585–8. doi: 10.1111/jth.15341 PMC8250233 34018298

[99] Apostolidis SA Sarkar A Giannini HM Goel RR Mathew D Suzuki A. Signaling through FcgammaRIIA and the C5a-C5aR pathway mediates platelet hyperactivation in COVID-19. bioRxiv (2021). doi: 10.1101/2021.05.01.442279 PMC8928747 35309299

[100] Nazy I Jevtic SD Moore JC Huynh A Smith JW Kelton JG. Platelet-activating immune complexes identified in critically ill COVID-19 patients suspected of heparin-induced thrombocytopenia. J Thromb Haemost (2021) 19:1342–7. doi: 10.1111/jth.15283 PMC8014456 33639037

[101] Reilly MP Taylor SM Franklin C Sachais BS Cines DB Williams KJ. Prothrombotic factors enhance heparin-induced thrombocytopenia and thrombosis *in vivo* in a mouse model. J Thromb Haemost (2006) 4:2687–94. doi: 10.1111/j.1538-7836.2006.02201.x 16961586

[102] Kerrigan SW Douglas I Wray A Heath J Byrne MF Fitzgerald D. A role for glycoprotein ib in *Streptococcus sanguis*-induced platelet aggregation. Blood (2002) 100:509–16. doi: 10.1182/blood.V100.2.509 12091342

[103] Sullam PM Hyun WC Szollosi J Dong J-F Foss WM Lopez JA. Physical proximity and functional interplay of the glycoprotein ib-IX-V complex and the fc receptor fcgamma RIIA on the platelet plasma membrane. J Biol Chem (1998) 273:5331–6. doi: 10.1074/jbc.273.9.5331 9478992

[104] Hally K Fauteux-Daniel S Hamzeh-Cognasse H Larsen P Cognasse F. Revisiting platelets and toll-like receptors (TLRs): At the interface of vascular immunity and thrombosis. Int J Mol Sci (2020) 21(17):6150. doi: 10.3390/ijms21176150 32858930 PMC7504402

[105] Cognasse F Nguyen KA Damien P Mcnicol A Pozzetto B Hamzeh-Cognasse H. The inflammatory role of platelets via their TLRs and siglec receptors. Front Immunol (2015) 6:83. doi: 10.3389/fimmu.2015.00083 25784910 PMC4345914

[106] Galgano L Guidetti GF Torti M Canobbio I. The controversial role of LPS in platelet activation *In vitro* . Int J Mol Sci (2022) 23(18):10900. doi: 10.3390/ijms231810900 36142813 PMC9505944

[107] Assinger A. Platelets and infection – an emerging role of platelets in viral infection. Front Immunol (2014) 5. doi: 10.3389/fimmu.2014.00649 PMC4270245 25566260

[108] Zheng M Karki R Williams EP Yang D Fitzpatrick E Vogel P. TLR2 senses the SARS-CoV-2 envelope protein to produce inflammatory cytokines. Nat Immunol (2021) 22:829–38. doi: 10.1038/s41590-021-00937-x PMC8882317 33963333

[109] Carnevale R Cammisotto V Bartimoccia S Nocella C Castellani V Bufano M. Toll-like receptor 4-dependent platelet-related thrombosis in SARS-CoV-2 infection. Circ Res (2023) 132:290–305. doi: 10.1161/CIRCRESAHA.122.321541 36636919

[110] Dib PRB Quirino-Teixeira AC Merij LB Pinheiro MBM Rozini SV Andrade FB. Innate immune receptors in platelets and platelet-leukocyte interactions. J Leukoc Biol (2020) 108:1157–82. doi: 10.1002/JLB.4MR0620-701R 32779243

[111] Marín Oyarzún CP Glembotsky AC Goette NP Lev PR De Luca G Baroni Pietto MC. Platelet toll-like receptors mediate thromboinflammatory responses in patients with essential thrombocythemia. Front Immunol (2020) 11:705. doi: 10.3389/fimmu.2020.00705 32425934 PMC7203216

[112] Banerjee M Huang Y Joshi S Popa GJ Mendenhall MD Wang QJ. Platelets endocytose viral particles and are activated via TLR (Toll-like receptor) signaling. Arterioscler Thromb Vasc Biol (2020) 40:1635–50. doi: 10.1161/ATVBAHA.120.314180 PMC7316618 32434410

[113] Rahimi N. C-type lectin CD209L/L-SIGN and CD209/DC-SIGN: cell adhesion molecules turned to pathogen recognition receptors. Biology (2020) 10:1. doi: 10.3390/biology10010001 33375175 PMC7822156

[114] Bauer B Steinle A. HemITAM: a single tyrosine motif that packs a punch. Sci Signal (2017) 10:eaan3676. doi: 10.1126/scisignal.aan3676 29208681

[115] Hottz ED Oliveira MF Nunes PCG Nogueira RMR Valls-De-Souza R Da Poian AT. Dengue induces platelet activation, mitochondrial dysfunction and cell death through mechanisms that involve DC-SIGN and caspases. J Thromb Haemost (2013) 11:951–62. doi: 10.1111/jth.12178 PMC3971842 23433144

[116] Tomo S Mohan S Ramachandrappa VS Samadanam DM Suresh S Pillai AB. Dynamic modulation of DC-SIGN and FcΥR2A receptors expression on platelets in dengue. PloS One (2018) 13:e0206346. doi: 10.1371/journal.pone.0206346 30412591 PMC6226166

[117] Londrigan SL Turville SG Tate MD Deng Y-M Brooks AG Reading PC. N-linked glycosylation facilitates sialic acid-independent attachment and entry of influenza a viruses into cells expressing DC-SIGN or l-SIGN. J Virol (2011) 85:2990–3000. doi: 10.1128/JVI.01705-10 21191006 PMC3067946

[118] Shih YP Chen CY Liu SJ Chen KH Lee YM Chao YC. Identifying epitopes responsible for neutralizing antibody and DC-SIGN binding on the spike glycoprotein of the severe acute respiratory syndrome coronavirus. J Virol (2006) 80:10315–24. doi: 10.1128/JVI.01138-06 PMC1641789 17041212

[119] Amraei R Yin W Napoleon MA Suder EL Berrigan J Zhao Q. CD209L/L-SIGN and CD209/DC-SIGN act as receptors for SARS-CoV-2. bioRxiv (2021) 2020. doi: 10.1101/2020.06.22.165803 PMC8265543 34341769

[120] Simpson JD Ray A Marcon C Dos Santos Natividade R Dorrazehi GM Durlet K. Single-molecule analysis of SARS-CoV-2 binding to c-type lectin receptors. Nano Lett (2023). doi: 10.1021/acs.nanolett.2c04931 PMC9924085 36758952

[121] Sung PS Hsieh SL. CLEC2 and CLEC5A: pathogenic host factors in acute viral infections. Front Immunol (2019) 10:2867. doi: 10.3389/fimmu.2019.02867 31867016 PMC6909378

[122] Heal JM Masel D Blumberg N. Interaction of platelet fc and complement receptors with circulating immune complexes involving the AB0 system. Vox Sang (1996) 71:205–11. doi: 10.1159/000462059 8958643

[123] Ford I Douglas CW Cox D Rees DG Heath J Preston FE. The role of immunoglobulin G and fibrinogen in platelet aggregation by *Streptococcus sanguis* . Br J Haematol (1997) 97:737–46. doi: 10.1046/j.1365-2141.1997.1342950.x 9217171

[124] Fletcher-Sandersjöö A Bellander B-M. Is COVID-19 associated thrombosis caused by overactivation of the complement cascade? a literature review. Thromb Res (2020) 194:36–41. doi: 10.1016/j.thromres.2020.06.027 32569879 PMC7301826

[125] Noris M Benigni A Remuzzi G. The case of complement activation in COVID-19 multiorgan impact. Kidney Int (2020) 98:314–22. doi: 10.1016/j.kint.2020.05.013 PMC7246017 32461141

[126] Yu J Yuan X Chen H Chaturvedi S Braunstein EM Brodsky RA. Direct activation of the alternative complement pathway by SARS-CoV-2 spike proteins is blocked by factor d inhibition. Blood (2020) 136:2080–9. doi: 10.1182/blood.2020008248 PMC7596849 32877502

[127] Atyeo C Fischinger S Zohar T Slein MD Burke J Loos C. Distinct early serological signatures track with SARS-CoV-2 survival. Immunity (2020) 53:524–532.e524. doi: 10.1016/j.immuni.2020.07.020 32783920 PMC7392190

[128] Hoepel W Chen H-J Geyer CE Allahverdiyeva S Manz XD De Taeye SW. High titers and low fucosylation of early human anti–SARS-CoV-2 IgG promote inflammation by alveolar macrophages. Sci Trans Med (2021) 13:eabf8654. doi: 10.1126/scitranslmed.abf8654 PMC8158960 33979301

[129] Bye AP Hoepel W Mitchell JL Jégouic S Loureiro S Sage T. Aberrant glycosylation of anti-SARS-CoV-2 IgG is a pro-thrombotic stimulus for platelets. bioRxiv (2021). doi: 10.1101/2021.03.26.437014 PMC8321687 34315173

[130] Kerrigan S Cox D. The effect of bacterial toxins on platelet function. In: Kini RM Clemetson KJ Markland FS Mclane MA Morita T, editors. Toxins and hemostasis from bench to bedside. Heidelberg: Springer (2010). p. 637–51.

[131] Binsker U Palankar R Wesche J Kohler TP Prucha J Burchhardt G. Secreted immunomodulatory proteins of staphylococcus aureus activate platelets and induce platelet aggregation. Thromb Haemost (2018) 118:745–57. doi: 10.1055/s-0038-1637735 29554697

[132] Vanassche T Kauskot A Verhaegen J Peetermans W Van Ryn J Schneewind O. Fibrin formation by staphylothrombin facilitates staphylococcus aureus -induced platelet aggregation. Thromb Haemost (2012) 107:1107–21. doi: 10.1160/TH11-12-0891 22437005

[133] Massini P Näf U. Ca2+ ionophores and the activation of human blood platelets: The effects of ionomycin, beauvericin, lysocellin, virginiamycin S, lasalocid-derivatives and McN 4308. Biochim Biophys Acta (1980) 598(3):575–87. doi: 10.1016/0005-2736(80)90037-1 6770901

[134] Landoni VI Pittaluga JR Carestia A Castillo LA Nebel MC Martire-Greco D. Neutrophil extracellular traps induced by shiga toxin and lipopolysaccharide-treated platelets exacerbate endothelial cell damage. Front Cell Infect Microbiol (2022) 12:897019. doi: 10.3389/fcimb.2022.897019 35811684 PMC9262415

[135] Hu H Armstrong PC Khalil E Chen YC Straub A Li M. GPVI and GPIbα mediate staphylococcal superantigen-like protein 5 (SSL5) induced platelet activation and direct toward glycans as potential inhibitors. PloS One (2011) 6(4):e19190. doi: 10.1371/journal.pone.0019190 21552524 PMC3084272

[136] Guo M Yi T Wang Q Wang D Feng P Kesheng D. TSST-1 protein exerts indirect effect on platelet activation and apoptosis. Platelets (2022) 33:998–1008. doi: 10.1080/09537104.2022.2026907 35073811

[137] Lourbakos A Potempa J Travis J D'andrea MR Andrade-Gordon P Santulli R. Arginine-specific protease from *Porphyromonas gingivalis* activates protease-activated receptors on human oral epithelial cells and induces interleukin-6 secretion. Infect Immun (2001) 69:5121–30. doi: 10.1128/IAI.69.8.5121-5130.2001 PMC98608 11447194

[138] Lourbakos A Yuan Y Jenkins AL Travis J Andrade-Gordon P Santulli R. Activation of protease-activated receptors by gingipains from *Porphyromonas gingivalis* leads to platelet aggregation: a new trait in microbial pathogenicity. Blood (2001) 97:3790–7. doi: 10.1182/blood.V97.12.3790 11389018

[139] Naito M Sakai E Shi Y Ideguchi H Shoji M Ohara N. *Porphyromonas gingivalis*-induced platelet aggregation in plasma depends on Hgp44 adhesin but not rgp proteinase. Mol Microbiol (2006) 59:152–67. doi: 10.1111/j.1365-2958.2005.04942.x 16359325

[140] Kerrigan SW Devine T Fitzpatrick G Thachil J Cox D. Early host interactions that drive the dysregulated response in sepsis. Front Immunol (2019) 10. doi: 10.3389/fimmu.2019.01748 PMC6691039 31447831

[141] Maneta E Aivalioti E Tual-Chalot S Emini Veseli B Gatsiou A Stamatelopoulos K. Endothelial dysfunction and immunothrombosis in sepsis. Front Immunol (2023) 14:1144229. doi: 10.3389/fimmu.2023.1144229 37081895 PMC10110956

[142] Garciarena CD Mchale TM Watkin RL Kerrigan SW. Coordinated molecular cross-talk between staphylococcus aureus, endothelial cells and platelets in bloodstream infection. Pathogens (2015) 4:869–82. doi: 10.3390/pathogens4040869 PMC4693168 26690226

[143] Rahbar A Soderberg-Naucler C. Human cytomegalovirus infection of endothelial cells triggers platelet adhesion and aggregation. J Virol (2005) 79:2211–20. doi: 10.1128/JVI.79.4.2211-2220.2005 PMC546536 15681423

[144] Espinola-Klein C Rupprecht H-J Blankenberg S Bickel C Kopp H Victor A. Impact of infectious burden on progression of carotid atherosclerosis. Stroke (2002) 33:2581–6. doi: 10.1161/01.STR.0000034789.82859.A4 12411646

[145] Hottz ED Bozza FA Bozza PT. Platelets in immune response to virus and immunopathology of viral infections. Front Med (2018) 5. doi: 10.3389/fmed.2018.00121 PMC5936789 29761104

[146] Papayannopoulos V. Neutrophil extracellular traps in immunity and disease. Nat Rev Immunol (2018) 18:134–47. doi: 10.1038/nri.2017.105 28990587

[147] Clark SR Ma AC Tavener SA Mcdonald B Goodarzi Z Kelly MM. Platelet TLR4 activates neutrophil extracellular traps to ensnare bacteria in septic blood. Nat Med (2007) 13:463–9. doi: 10.1038/nm1565 17384648

[148] Jiao Y Li W Wang W Tong X Xia R Fan J. Platelet-derived exosomes promote neutrophil extracellular trap formation during septic shock. Crit Care (2020) 24:380. doi: 10.1186/s13054-020-03082-3 32600436 PMC7322900

[149] Gomez RM Lopez Ortiz AO Schattner M. Platelets and extracellular traps in infections. Platelets (2021) 32:305–13. doi: 10.1080/09537104.2020.1718631 31984825

[150] Marx C Novotny J Salbeck D Zellner KR Nicolai L Pekayvaz K. Eosinophil-platelet interactions promote atherosclerosis and stabilize thrombosis with eosinophil extracellular traps. Blood (2019) 134:1859–72. doi: 10.1182/blood.2019000518 PMC6908806 31481482

[151] Sim MS Kim HJ Bae I Kim C Chang HS Choi Y. Calcium ionophore-activated platelets induce eosinophil extracellular trap formation. Allergol Int (2022). doi: 10.1016/j.alit.2022.12.002 36586745

[152] Fu G Deng M Neal MD Billiar TR Scott MJ. Platelet-monocyte aggregates: understanding mechanisms and functions in sepsis. Shock (2021) 55:156–66. doi: 10.1097/SHK.0000000000001619 PMC8008955 32694394

[153] Gautam I Huss CW Storad ZA Krebs M Bassiouni O Ramesh R. Activated platelets mediate monocyte killing of klebsiella pneumoniae. Infect Immun (2023) 91:e0055622. doi: 10.1128/iai.00556-22 36853027 PMC10016073

[154] Hottz ED Azevedo-Quintanilha IG Palhinha L Teixeira L Barreto EA Pão CRR. Platelet activation and platelet-monocyte aggregate formation trigger tissue factor expression in patients with severe COVID-19. Blood (2020) 136:1330–41. doi: 10.1182/blood.2020007252 PMC7483437 32678428

[155] Hottz ED Medeiros-De-Moraes IM Vieira-De-Abreu A De Assis EF Vals-De-Souza R Castro-Faria-Neto HC. Platelet activation and apoptosis modulate monocyte inflammatory responses in dengue. J Immunol (2014) 193:1864–72. doi: 10.4049/jimmunol.1400091 PMC4137323 25015827

[156] Taus F Salvagno G Cane S Fava C Mazzaferri F Carrara E. Platelets promote thromboinflammation in SARS-CoV-2 pneumonia. Arterioscler Thromb Vasc Biol (2020) 40:2975–89. doi: 10.1161/ATVBAHA.120.315175 PMC7682791 33052054

[157] Singh A Bisht P Bhattacharya S Guchhait P. Role of platelet cytokines in dengue virus infection. Front Cell Infect Microbiol (2020) 10:561366–6. doi: 10.3389/fcimb.2020.561366 PMC7554584 33102253

[158] Alonso AL Cox D. Platelet interactions with viruses and parasites. Platelets (2015) 26:317–23. doi: 10.3109/09537104.2015.1025376 25915557

[159] Seyoum M Enawgaw B Melku M. Human blood platelets and viruses: defense mechanism and role in the removal of viral pathogens. Thromb J (2018) 16:16. doi: 10.1186/s12959-018-0170-8 30026673 PMC6048695

[160] Antoniak S Mackman N. Platelets and viruses. Platelets (2021) 32:325–30. doi: 10.1080/09537104.2021.1887842 PMC7987802 33615982

[161] Schrottmaier WC Schmuckenschlager A Pirabe A Assinger A. Platelets in viral infections - brave soldiers or Trojan horses. Front Immunol (2022) 13:856713. doi: 10.3389/fimmu.2022.856713 35419008 PMC9001014

[162] Simmons CP Farrar JJ Van Vinh Chau N Wills B. Dengue. N Engl J Med (2012) 366:1423–32. doi: 10.1056/NEJMra1110265 22494122

[163] Harapan H Michie A Sasmono RT Imrie A. Dengue: a minireview. Viruses (2020) 12(8):829. doi: 10.3390/v12080829 32751561 PMC7472303

[164] Gerber MA Baltimore RS Eaton CB Gewitz M Rowley AH Shulman ST. Prevention of rheumatic fever and diagnosis and treatment of acute streptococcal pharyngitis. a scientific statement from the American heart association rheumatic fever, endocarditis, and Kawasaki disease committee of the council on cardiovascular disease in the young, the interdisciplinary council on functional genomics and translational biology, and the interdisciplinary council on quality of care and outcomes research. Circulation (2009). doi: 10.1161/CIRCULATIONAHA.109.191959 19246689

[165] Hoen B Duval X. Infective endocarditis. N Engl J Med (2013) 368:1425–33. doi: 10.1056/NEJMcp1206782 23574121

[166] Chambers HF Bayer AS. Native-valve infective endocarditis. N Engl J Med (2020) 383:567–76. doi: 10.1056/NEJMcp2000400 32757525

[167] Charlesworth M Williams BG Ray S. Infective endocarditis. BJA Educ (2023) 23:144–52. doi: 10.1016/j.bjae.2023.01.001 PMC10028394 36960439

[168] Kerrigan SW Cox D. Platelet-bacterial interactions as therapeutic targets in infective endocarditis. In: Endocarditis, edited by Breijo-Márque FR. InTech (2012) 51–74. doi: 10.5772/1192

[169] Leeten K Jacques N Lancellotti P Oury C. Aspirin or ticagrelor in staphylococcus aureus infective endocarditis: where do we stand? Front Cell Dev Biol (2021) 9:716302. doi: 10.3389/fcell.2021.716302 34692677 PMC8529053

[170] Polzin A Dannenberg L M'pembele R Mourikis P Naguib D Zako S. Staphylococcus aureus increases platelet reactivity in patients with infective endocarditis. Sci Rep (2022) 12:12933. doi: 10.1038/s41598-022-16681-7 35902612 PMC9334290

[171] Yeaman MR. Platelets: At the nexus of antimicrobial defence. Nat Rev Micro (2014) 12:426–37. doi: 10.1038/nrmicro3269 24830471

[172] Sun H Wang X Degen JL Ginsburg D. Reduced thrombin generation increases host susceptibility to group a streptococcal infection. Blood (2009) 113:1358–64. doi: 10.1182/blood-2008-07-170506 PMC2637198 19056689

[173] Beristain-Covarrubias N Perez-Toledo M Flores-Langarica A Zuidscherwoude M Hitchcock JR Channell WM. Salmonella-induced thrombi in mice develop asynchronously in the spleen and liver and are not effective bacterial traps. Blood (2019) 133:600–4. doi: 10.1182/blood-2018-08-867267 PMC6474721 30401709

[174] Hitchcock JR Cook CN Bobat S Ross EA Flores-Langarica A Lowe KL. Inflammation drives thrombosis after salmonella infection via CLEC-2 on platelets. J Clin Invest (2015) 125:4429–46. doi: 10.1172/JCI79070 PMC4665792 26571395

[175] Rayes J Lax S Wichaiyo S Watson SK Di Y Lombard S. The podoplanin-CLEC-2 axis inhibits inflammation in sepsis. Nat Commun (2017) 8:2239. doi: 10.1038/s41467-017-02402-6 29269852 PMC5740111

[176] Claushuis T De Vos AF Nieswandt B Boon L Roelofs J De Boer OJ. Platelet glycoprotein VI aids in local immunity during pneumonia-derived sepsis caused by gram-negative bacteria. Blood (2018) 131:864–76. doi: 10.1182/blood-2017-06-788067 29187378

[177] Kahn F Hurley S Shannon O. Platelets promote bacterial dissemination in a mouse model of streptococcal sepsis. Microbes Infect (2013) 15:669–76. doi: 10.1016/j.micinf.2013.05.003 23711899

[178] Rabouël Y Magnenat S Lefebvre F Delabranche X Gachet C Hechler B. Transfusion of fresh washed platelets does not prevent experimental polymicrobial-induced septic shock in mice. J Thromb Haemost (2022) 20:449–60. doi: 10.1111/jth.15583 34752015

[179] De Stoppelaar SF Van 'T Veer C Claushuis TA Albersen BJ Roelofs JJ van der Poll T. Thrombocytopenia impairs host defense in gram-negative pneumonia-derived sepsis in mice. Blood (2014) 124:3781–90. doi: 10.1182/blood-2014-05-573915 PMC4263985 25301709

[180] Van Den Boogaard FE Schouten M De Stoppelaar SF Roelofs JJTH Brands X Schultz MJ. Thrombocytopenia impairs host defense during murine streptococcus pneumoniae pneumonia. Crit Care Med (2015) 43:e75–83. doi: 10.1097/CCM.0000000000000853 25627210

[181] Wuescher LM Takashima A Worth RG. A novel conditional platelet depletion mouse model reveals the importance of platelets in protection against staphylococcus aureus bacteremia. J Thromb Haemost (2015) 13:303–13. doi: 10.1111/jth.12795 PMC4320667 25418277

[182] Halushka PV Wise WC Cook JA. Protective effects of aspirin in endotoxic shock. J Pharmacol Exp Ther (1981) 218:464–9. 6894770

[183] Halushka PV Wise WC Cook JA. Studies on the beneficial effects of aspirin in endotoxic shock: relationship to inhibition of arachidonic acid metabolism. Am J Med (1983) 74:91–6. doi: 10.1016/0002-9343(83)90535-1 6687979

[184] Carestia A Davis RP Grosjean H Lau MW Jenne CN. Acetylsalicylic acid inhibits intravascular coagulation during staphylococcus aureus-induced sepsis in mice. Blood (2020) 135:1281–6. doi: 10.1182/blood.2019002783 31951648

[185] Seidel M Winning J Claus RA Bauer M Lösche W. Beneficial effect of clopidogrel in a mouse model of polymicrobial sepsis. J Thromb Haemost (2009) 7:1030–2. doi: 10.1111/j.1538-7836.2009.03352.x 19548910

[186] Luo Q Liu R Qu K Liu G Hang M Chen G. Cangrelor ameliorates CLP-induced pulmonary injury in sepsis by inhibiting GPR17. Eur J Med Res (2021) 26:70. doi: 10.1186/s40001-021-00536-4 34229761 PMC8262027

[187] Rabouel Y Magnenat S Delabranche X Gachet C Hechler B. Platelet P2Y (12) receptor deletion or pharmacological inhibition does not protect mice from sepsis or septic shock. TH Open (2021) 5(3):e343–52. doi: 10.1055/s-0041-1733857 PMC8384481 34447900

[188] Pulavendran S Rudd JM Maram P Thomas PG Akhilesh R Malayer JR. Combination therapy targeting platelet activation and virus replication protects mice against lethal influenza pneumonia. Am J Respir Cell Mol Biol (2019) 61:689–701. doi: 10.1165/rcmb.2018-0196OC 31070937 PMC6890408

[189] Hsu J Donnelly JP Chaudhary NS Moore JX Safford MM Kim J. Aspirin use and long-term rates of sepsis: a population-based cohort study. PloS One (2018) 13:e0194829. doi: 10.1371/journal.pone.0194829 29668690 PMC5905958

[190] Eisen DP Leder K Woods RL Lockery JE Mcguinness SL Wolfe R. Effect of aspirin on deaths associated with sepsis in healthy older people (ANTISEPSIS): a randomised, double-blind, placebo-controlled primary prevention trial. Lancet Respir Med (2021) 9:186–95. doi: 10.1016/S2213-2600(20)30411-2 PMC7957956 32950072

[191] Hsu WT Porta L Chang IJ Dao QL Tehrani BM Hsu TC. Association between aspirin use and sepsis outcomes: a national cohort study. Anesth Analg (2022) 135:110–7. doi: 10.1213/ANE.0000000000005943 35245223

[192] Lu Z Fang P Xia D Li M Li S Wang Y. The impact of aspirin exposure prior to intensive care unit admission on the outcomes for patients with sepsis-associated acute respiratory failure. Front Pharmacol (2023) 14:1125611. doi: 10.3389/fphar.2023.1125611 36937880 PMC10014538

[193] Rögnvaldsson KG Bjarnason A Kristinsson K Bragason HT Erlendsdóttir H Þorgeirsson G. Acetylsalicylic acid use is associated with improved survival in bacteremic pneumococcal pneumonia: a long-term nationwide study. J Intern Med (2022) 292:321–32. doi: 10.1111/joim.13485 PMC9543431 35315156

[194] Lavie I Lavie M Gafter-Gvili A Halperin E Abramovich-Yoffe H Avni T. Chronic aspirin use and survival following sepsis-a propensity-matched, observational cohort study. Clin Microbiol Infect (2022) 28:1287.e1281–1287.e1287. doi: 10.1016/j.cmi.2022.04.010 35533971

[195] Du F Jiang P He S Song D Xu F. Antiplatelet therapy for critically ill patients: a pairwise and Bayesian network meta-analysis. Shock (2018) 49:616–24. doi: 10.1097/SHK.0000000000001057 29176404

[196] Trauer J Muhi S Mcbryde ES Al Harbi SA Arabi YM Boyle AJ. Quantifying the effects of prior acetyl-salicylic acid on sepsis-related deaths: an individual patient data meta-analysis using propensity matching. Crit Care Med (2017) 45:1871–9. doi: 10.1097/CCM.0000000000002654 PMC5640482 28799949

[197] Sipila PN Lindbohm JV David Batty G Heikkila N Vahtera J Suominen S. Severe infection and risk of cardiovascular disease: a multicohort study. Circulation (2023). doi: 10.1161/CIRCULATIONAHA.122.061183 36971007

[198] Wei K-C Sy C-L Wang W-H Wu C-L Chang S-H Huang Y-T. Major acute cardiovascular events after dengue infection–a population-based observational study. PloS Negl Trop Dis (2022) 16:e0010134. doi: 10.1371/journal.pntd.0010134 35130277 PMC8853534

[199] De Groot B Van Den Berg S Kessler J Ansems A Rijpsma D. Independent predictors of major adverse cardiovascular events in emergency department patients who are hospitalised with a suspected infection: a retrospective cohort study. BMJ Open (2016) 6:e009598. doi: 10.1136/bmjopen-2015-009598 PMC4735138 26817637

[200] Tessitore E Carballo D Poncet A Perrin N Follonier C Assouline B. Mortality and high risk of major adverse events in patients with COVID-19 and history of cardiovascular disease. Open Heart (2021) 8:e001526. doi: 10.1136/openhrt-2020-001526 33833064 PMC8039226

[201] Aubin E Lemieux R Bazin R. Indirect inhibition of *in vivo* and *in vitro* T-cell responses by intravenous immunoglobulins due to impaired antigen presentation. Blood (2010) 115:1727–34. doi: 10.1182/blood-2009-06-225417 19965673

[202] Aggarwal R Dewan A Pandey A Trehan N Majid MA. Efficacy of high-dose intravenous immunoglobulin in severe and critical COVID-19: a retrospective cohort study. Int Immunopharmacol (2022) 106:108615. doi: 10.1016/j.intimp.2022.108615 35168081 PMC8825318

[203] Yang Y Yu X Zhang F Xia Y. Evaluation of the effect of intravenous immunoglobulin dosing on mortality in patients with sepsis: a network meta-analysis. Clin Ther (2019) 41:1823–1838.e1824. doi: 10.1016/j.clinthera.2019.06.010 31470986

[204] Group IC Brocklehurst P Farrell B King A Juszczak E Darlow B. Treatment of neonatal sepsis with intravenous immune globulin. N Engl J Med (2011) 365:1201–11. doi: 10.1056/NEJMoa1100441 21962214

[205] Rizvi MQ Singh MV Mishra N Shrivastava A Maurya M Siddiqui SA. Intravenous immunoglobulin in the management of neonatal sepsis: a randomised controlled trial. Trop Doctor (2023) 53:222–6. doi: 10.1177/00494755221138689 36654494

[206] Mccrindle BW Rowley AH Newburger JW Burns JC Bolger AF Gewitz M. Diagnosis, treatment, and long-term management of Kawasaki disease: a scientific statement for health professionals from the American heart association. Circulation (2017) 135:e927–99. doi: 10.1161/CIR.0000000000000484 28356445

[207] Rockman S Lowther S Camuglia S Vandenberg K Taylor S Fabri L. Intravenous immunoglobulin protects against severe pandemic influenza infection. EBioMedicine (2017) 19:119–27. doi: 10.1016/j.ebiom.2017.04.010 PMC5440604 28408242

[208] Cao W Liu X Hong K Ma Z Zhang Y Lin L. High-dose intravenous immunoglobulin in severe coronavirus disease 2019: a multicenter retrospective study in China. Front Immunol (2021) 12:627844. doi: 10.3389/fimmu.2021.627844 33679771 PMC7933558

[209] Pietersz GA Mottram PL Van De Velde NC Sardjono CT Esparon S Ramsland PA. Inhibition of destructive autoimmune arthritis in FcgammaRIIa transgenic mice by small chemical entities. Immunol Cell Biol (2009) 87:3–12. doi: 10.1038/icb.2008.82 19030019

[210] Mai SH Khan M Dwivedi DJ Ross CA Zhou J Gould TJ. Delayed but not early treatment with DNase reduces organ damage and improves outcome in a murine model of sepsis. Shock (2015) 44:166–72. doi: 10.1097/SHK.0000000000000396 26009820

[211] Lauková L Bertolo EMJ Zelinková M Borbélyová V Čonka J Gaál Kovalčíková A. Early dynamics of plasma dna in a mouse model of sepsis. Shock (2019) 52:257–63. doi: 10.1097/SHK.0000000000001215 30052582

[212] Shen X Cao K Zhao Y Du J. Targeting neutrophils in sepsis: from mechanism to translation. Front Pharmacol (2021) 12:644270. doi: 10.3389/fphar.2021.644270 33912055 PMC8072352

[213] Luo J Zhang Z Zhao S Gao R. A comparison of etiology, pathogenesis, vaccinal and antiviral drug development between influenza and COVID-19. Int J Mol Sci (2023) 24:6369. doi: 10.3390/ijms24076369 37047339 PMC10094131

[214] Basler CF. Molecular pathogenesis of viral hemorrhagic fever. Semin Immunopathol (2017) 39:551–61. doi: 10.1007/s00281-017-0637-x PMC6436832 28555386

[215] Banerjee G Shokeen K Chakraborty N Agarwal S Mitra A Kumar S. Modulation of immune response in Ebola virus disease. Curr Opin Pharmacol (2021) 60:158–67. doi: 10.1016/j.coph.2021.07.004 34425392

[216] Woolsey C Fears AC Borisevich V Agans KN Dobias NS Prasad AN. Natural history of Sudan ebolavirus infection in rhesus and cynomolgus macaques. Emerg Microbes Infect (2022) 11:1635–46. doi: 10.1080/22221751.2022.2086072 PMC9225728 35657325

[217] Gupta S Sakhuja A Kumar G Mcgrath E Nanchal RS Kashani KB. Culture-negative severe sepsis: nationwide trends and outcomes. Chest (2016) 150:1251–9. doi: 10.1016/j.chest.2016.08.1460 27615024

